# Attachment of Enterohemorrhagic Escherichia coli to Host Cells Reduces O Antigen Chain Length at the Infection Site That Promotes Infection

**DOI:** 10.1128/mBio.02692-21

**Published:** 2021-12-14

**Authors:** Bin Liu, Chengqian Qian, Pan Wu, Xiaodan Li, Yutao Liu, Huiqian Mu, Min Huang, Yang Zhang, Tianyuan Jia, Yuanyuan Wang, Lu Wang, Xiao Zhang, Di Huang, Bin Yang, Lu Feng, Lei Wang

**Affiliations:** a TEDA Institute of Biological Sciences and Biotechnology, Nankai University, Tianjin, People’s Republic of China; b The Institute of Translational Medicine Research, Tianjin Union Medical Center, Nankai University Affiliated Hospital, Nankai University, Tianjin, People’s Republic of China; c The Key Laboratory of Molecular Microbiology and Technology, Ministry of Education, Tianjin, People’s Republic of China; d Tianjin Key Laboratory of Microbial Functional Genomics, Tianjin, People’s Republic of China; e State Key Laboratory of Medicinal Chemical Biology, Nankai University, Tianjin, People’s Republic of China

**Keywords:** enterohemorrhagic *Escherichia coli*, type III secretion system, O antigen chain length, virulence, epithelial cell attachment

## Abstract

Many enteropathogenic bacteria express a needle-like type III secretion system (T3SS) that translocates effectors into host cells promoting infection. O antigen (OAg) constitutes the outer layer of Gram-negative bacteria protecting bacteria from host immune responses. *Shigella* constitutively shortens the OAg molecule in its three-dimensional conformation by glucosylation, leading to enhanced T3SS function. However, whether and how other enteropathogenic bacteria shorten the OAg molecule that probably facilitates infection remain unknown. For the first time, we report a smart mechanism by which enterohemorrhagic Escherichia coli specifically reduces the size of the OAg molecule at the infection site upon sensing mechanical signals of intestinal epithelial cell attachment via the membrane protein YgjI. YgjI represses expression of the OAg chain length regulator gene *fepE* via the global regulator H-NS, leading to shortened OAg chains and injection of more T3SS effectors into host cells. However, bacteria express long-chain OAg in the intestinal lumen benefiting their survival. Animal experiments show that blocking this regulatory pathway significantly attenuates bacterial virulence. This finding enhances our understanding of interactions between the surfaces of bacterial and host cells and the way this interaction enhances bacterial pathogenesis.

## INTRODUCTION

Enteropathogenic bacteria, such as pathogenic Escherichia coli, Salmonella, and *Shigella*, cause severe-to-moderate infectious gastroenteritis, which results in approximately 300 million cases of illness annually worldwide (1, 2). After ingestion by hosts, enteropathogenic bacteria must first travel through the gastrointestinal tract to the infection site (small or large intestine) while resisting components of host defense systems, such as gastric acid and different innate immune factors (3, 4). Among these factors, antimicrobial peptides are fundamental components of the innate immune system that are inserted into the bacterial membrane and cause pore formation, resulting in leakage of cellular contents, disruption of membrane functions, and lysis of bacterial cells (5, 6).

At the infection site, enteropathogenic bacteria establish infection by attaching to and/or invading intestinal epithelial cells (2). During infection of epithelial cells, many enteropathogenic bacteria, including Salmonella and pathogenic E. coli, express a type III secretion system (T3SS) through which bacterial effectors required for cell attachment, cell invasion, and subversion of host cell processes are translocated into the cytosol of host intestinal epithelial cells (7). T3SS mutations in these bacteria lead to the attenuation of bacterial virulence (8, 9). A functional T3SS contains a basal body embedded in the bacterial inner and outer membranes, a needle-like oligomeric structure that protrudes from the outer membrane, and a translocation pore inserted into the target cell membrane and linked with the T3SS needle (10). The translocation pore and T3SS needle establish a conduit between the bacterial cytoplasm and host cell cytosol, which enables the direct delivery of effectors (11).

Besides T3SS, enteropathogenic bacteria contain several factors that facilitate the infection process. For instance, various lipopolysaccharide (LPS) components, such as O antigen (OAg) or O-polysaccharide, have been shown to be required for the resistance of pathogenic bacteria to antimicrobial peptides *in vitro* (12–14). OAg forms the outermost component of LPS in Gram-negative bacteria and is the major constituent of the outer leaflet of the outer membrane; it extends outward from the bacterial surface and is in direct contact with the environment that surrounds the bacterial cell (10). OAg consists of oligosaccharide repeats (O-units), and it can present in two different lengths of approximately 15 O-units (short OAg chains) and >80 O-units (long OAg chains). In E. coli, the synthesis of short and long OAg chains are positively regulated by Wzz and FepE, respectively (15, 16).

Several studies have demonstrated that short OAg chains benefit T3SS effector injection, possibly through enhancing the access of T3SS to host cells. For instance, Salmonella Δ*fepE* mutant with short OAg chains exhibits increased translocation of effectors secreted by the T3SS encoded by Salmonella pathogenicity island 1 (SPI1) and enhanced Salmonella invasion of epithelial cells *in vitro* (17). *Shigella* constitutively shortens the OAg molecule in its three-dimensional conformation via glucosylation, which optimizes the exposure of T3SS needles, leading to increased bacterial invasion *in vitro* and enhanced bacterial colonization *in vivo* (18). However, long OAg chains benefit bacterial survival in the intestinal lumen by increasing bacterial resistance to innate immune factors, such as antimicrobial peptides (17, 19). It remains unknown whether and how EHEC bacteria promote the function of T3SS by specially reducing OAg chain length upon attaching to the intestinal epithelium.

Enterohemorrhagic E. coli (EHEC) is an important human gastrointestinal pathogen that causes bloody diarrhea and can result in hemolytic uremic syndrome that is fatal in 3 to 75% of cases (20). EHEC specifically colonizes the epithelium of the large intestine (21). The process of EHEC attachment to intestinal epithelial cells can be divided into two major steps (22): (i) initial attachment mediated by the binding of bacterial adhesion factors to their receptors on the surface of host epithelial cells (23) and (ii) intimate attachment mediated by the EHEC T3SS and secreted effectors (24).

In this study, we used EHEC O157:H7 strain EDL933 as a model to investigate the regulation of bacterial OAg chain length in the intestinal tract, the underlying molecular mechanisms, and the relationship of OAg chain length with bacterial survival and pathogenicity.

## RESULTS

### HeLa-attached EHEC presents shorter OAg chains than unattached bacterial cells.

**FIG 1:**
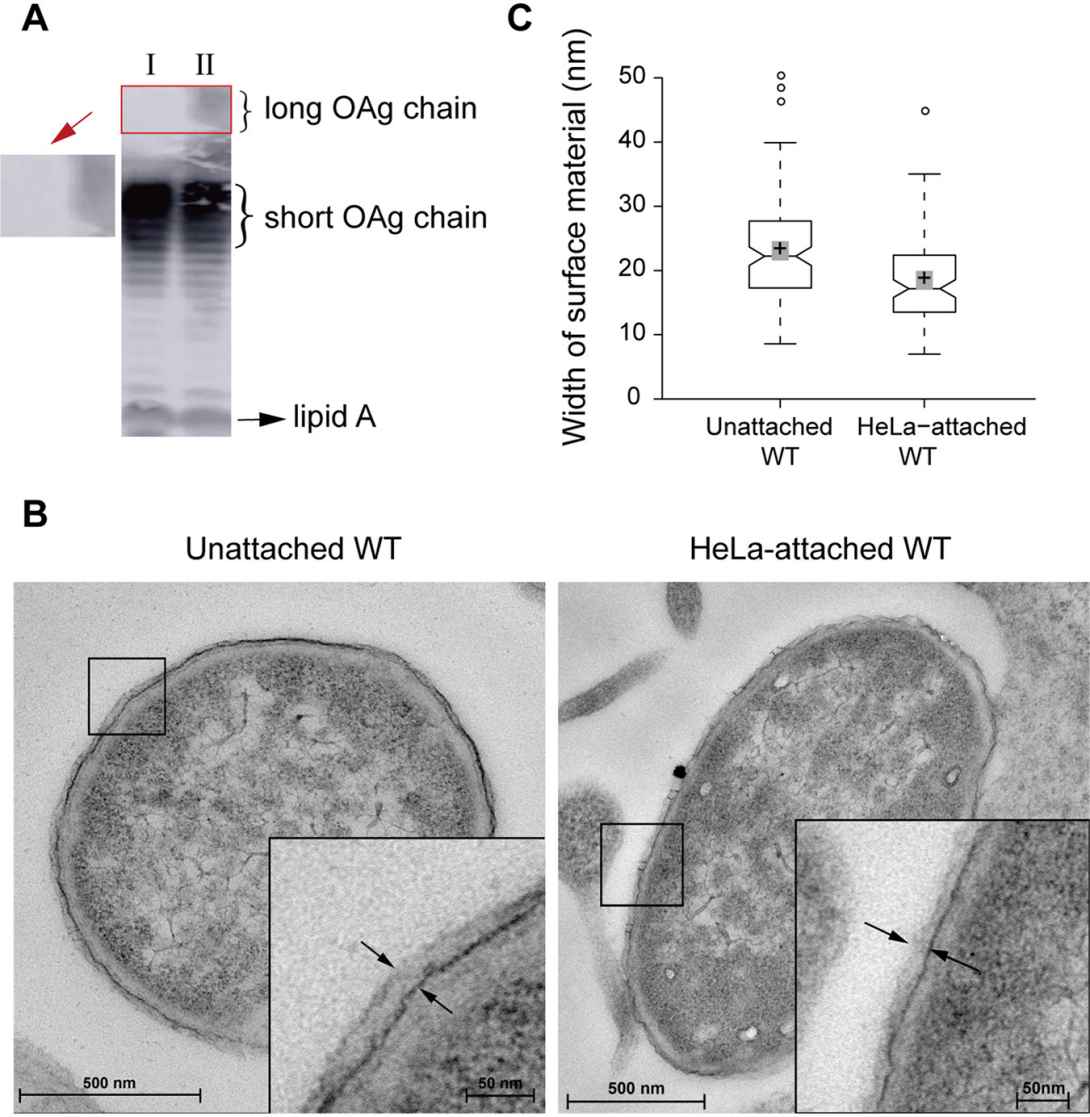
EHEC strain EDL933 exhibits decreased OAg chain length when attached to HeLa cells. (A) Western blotting of LPS obtained from HeLa-attached EDL933 (I) and unattached EDL933 (II) using anti-O157 monoclonal antiserum. (B and C) Surface topology of unattached EDL933 (wild type [WT]) cells and EDL933 (WT) cells attached to HeLa cells as visualized by TEM. The electron-dense material (LPS) at the bacterial surface is demarcated by the two opposing arrows in the insets. The regions within the boxes show magnified views. (C) In the box plots, center lines show the medians, the box lines indicate the 25th and 75th percentiles as determined by R software. The whiskers extend 1.5 times the interquartile range from the 25th and 75th percentiles, with outliers represented by dots. The crosses represent data means, and the gray bars indicate 95% confidence intervals of the means. Notches in the boxes emanating from the median values represent the 95% confidence interval for the median values. When the notches of different boxes do not overlap, the medians are considered significantly different (61, 62). The average widths of the material extending from the outer membrane in unattached and HeLa-attached EDL933 cells were 23.1 nm and 18.5 nm, respectively. The data are representative of 10 cells from three independent experiments.

To investigate whether the OAg chain length is regulated in response to host cell attachment, LPS prepared from EHEC strain EDL933 attached to HeLa cells and unattached EDL933 was analyzed by sodium dodecyl sulfate-polyacrylamide gel electrophoresis (SDS-PAGE) and Western blotting. The production of long OAg chains on strain EDL933 attached to HeLa cells was reduced compared to that on unattached EDL933 (Fig. 1A). In addition, transmission electron microscopy (TEM) was used to determine the average width of the electron-dense surface material that extended from the outer membrane, which is mainly comprised of LPS material. The average width of LPS material was 23.1 nm in unattached EDL933 cells but 18.5 nm in HeLa-attached EDL933 cells, a decrease of 19.9% (Fig. 1B and C). EHEC O157 also produces a group 4 capsule (G4C), whose assembly is dependent on Etp (25). To exclude the possibility that the increase in the width of the electron-dense surface material that extended from the outer membrane on unattached EDL933 was due to changes in G4C rather than changes in LPS, we compared the width of the outer material on unattached EDL933 and EDL933Δ*etp* mutant using TEM analysis and found that the width of the material extending from the outer membrane of these two strains exhibited no significant difference (see Fig. S1 in the supplemental material). In contrast, deletion of *waaL*, which is responsible for the linkage of OAg to the lipid A-core oligosaccharide complex during LPS synthesis (26), reduced the average width of the material extending from the bacterial outer membrane on unattached bacteria to 16.4 nm (Fig. S1). This finding confirms that the material extending from the outer membrane of strain EDL933 is an LPS constituent, not the G4C. These data indicate that the OAg chain length is reduced when EDL933 attaches to HeLa cells.

10.1128/mBio.02692-21.1 FIG S1 Surface topology of the EDL933Δ*etp*(Δ*etp*) and EDL933Δ*waaL*(Δ*waaL*) mutants as visualized by TEM. The electron-dense material extending from the bacterial surface is demarcated by the two opposing arrows. The boxed areas represent magnified views. In the box plots, center lines show the medians, and the box lines indicate the 25th and 75th percentiles as determined by R software. Whiskers extend 1.5 times the interquartile range from the 25th and 75th percentiles, with outliers represented by dots. Crosses represent data means, and gray bars indicate 95% confidence intervals of the means. Notches in the boxes emanating from the median values represent the 95% confidence interval for the median values. When the notches of different boxes do not overlap, the medians are considered significantly different. The average widths of material extending from the outer membrane in the EDL933Δ*etp* (Δ*etp*) and EDL933Δ*waaL* (Δ*waaL*) strains were 23.8 nm and 16.4 nm, respectively. The data are representative of 10 cells from three independent experiments. WT denotes wild-type EDL933. Download FIG S1, TIF file, 1.7 MB. Copyright © 2021 Liu et al. 2021 Liu et al. https://creativecommons.org/licenses/by/4.0/ This content is distributed under the terms of the Creative Commons Attribution 4.0 International license.

### *fepE* expression is downregulated following attachment of EHEC to host cells *in vitro* and *in vivo*, leading to a reduction of the OAg chain length.

**FIG 2:**
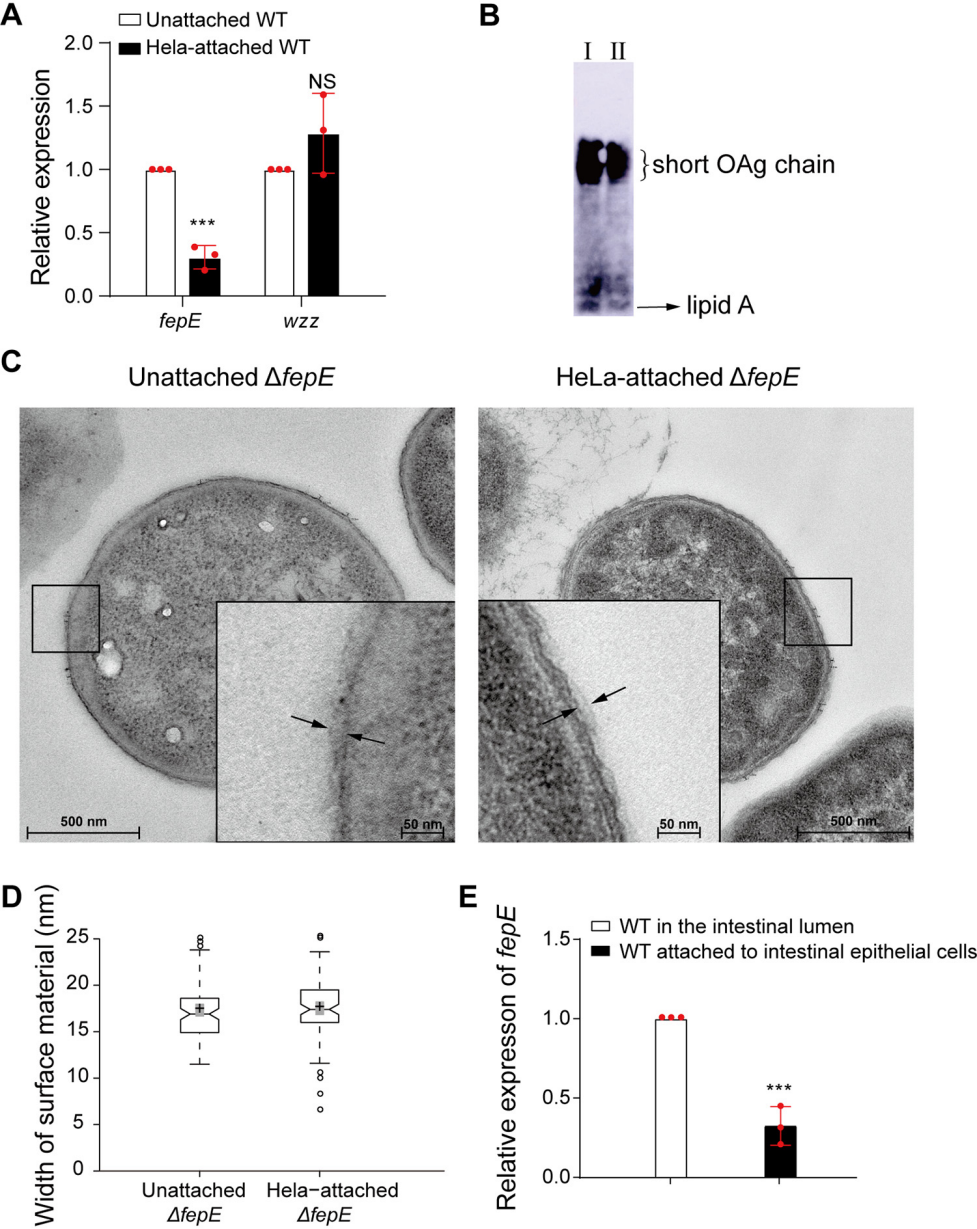
FepE is responsible for the reduction in OAg chain length following the attachment of strain EDL933 to HeLa cells. (A) Relative expression of *fepE* and *wzz* in HeLa-attached and unattached EDL933 (WT) as determined by qRT-PCR. The error bars represent standard deviations (SD). Each experiment was replicated three times independently. (B) Western blotting of LPS obtained from HeLa-attached EDL933Δ*fepE* (I) and unattached EDL933Δ*fepE* cells (II) using anti-O157 monoclonal antiserum. (C and D) Surface topology of HeLa-attached EDL933Δ*fepE* (Δ*fepE*) and unattached EDL933Δ*fepE* (Δ*fepE*) as visualized by TEM. The electron-dense material at the bacterial surface is demarcated by the two opposing arrows in the insets. The regions within the boxes show magnified views. In the box plots, the center lines show the medians, and box lines indicate the 25th and 75th percentiles as determined by R software. Whiskers extend 1.5 times the interquartile range from the 25th and 75th percentiles, with outliers represented by dots. Crosses represent data means, and gray bars indicate 95% confidence intervals of the means. Notches in the boxes emanating from the median values represent the 95% confidence interval for the median values. When the notches of different boxes overlap, the medians are considered not significantly different (61, 62). The average widths of material extending from the outer membrane in unattached and HeLa-attached Δ*fepE* cells were 17.3 nm and 17.5 nm, respectively. The data are representative of 10 cells from three independent experiments. (E) Relative expression of *fepE* expression in EDL933 (WT) cells containing pLW*-fepE*p-*gfp* in the intestinal epithelium and lumen of infant rabbits as determined by qRT-PCR. The constitutively expressed *rfp* gene inserted into the EDL933 chromosome was used as the reference control for normalization. The error bars represent SD. Each experiment was replicated three times independently. ***, *P* < 0.001; NS, not significant.

To investigate whether FepE and/or Wzz, which regulate the synthesis of long and short OAg chains (15, 16), respectively, are involved in the regulation of OAg chain length in response to host cell attachment, we first analyzed the expression of the genes encoding these two proteins in HeLa-attached and unattached EDL933. The *fepE* expression levels in HeLa-attached EDL933 were decreased by 3.3-fold compared with that in unattached EDL933 (Fig. 2A). In contrast, the *wzz* expression levels were not significantly different between HeLa-attached and unattached EDL933 (Fig. 2A), suggesting that *fepE* expression rather than *wzz* is involved in the regulation of OAg chain length under these conditions. The same result (downregulation of *fepE* in attached EDL933) was also obtained when Caco-2 intestinal epithelial cells were used instead of HeLa cells (Fig. S2A).

10.1128/mBio.02692-21.2 FIG S2 Relative expression of *fepE* in EHEC and EPEC strains when attached to Caco-2 cells (A) or HeLa cells (B). Download FIG S2, TIF file, 2.2 MB. Copyright © 2021 Liu et al. 2021 Liu et al. https://creativecommons.org/licenses/by/4.0/ This content is distributed under the terms of the Creative Commons Attribution 4.0 International license.

To investigate how *fepE* downregulation affects OAg chain length, we compared the LPS patterns in HeLa-attached EDL933Δ*fepE* mutant and unattached EDL933Δ*fepE* mutant using SDS-PAGE and Western blot analyses and found that their LPS patterns were the same, both producing only short OAg chains (Fig. 2B). The results of TEM analysis indicated that the width of the material extending from the outer membrane of the HeLa-attached and unattached EDL933Δ*fepE* mutant was not significantly different and that the width was notably smaller on the mutant strain (17.3 nm on average) than on the unattached wild-type strain (Fig. 2C and D). These findings suggest that the reduction in *fepE* expression upon EDL933 attachment to HeLa cells results in the reduced OAg chain length.

To investigate whether *fepE* downregulation also occurs *in vivo* upon attachment to intestinal epithelial cells, we constructed a plasmid containing the *fepE* promoter transcriptionally fused to a green fluorescent protein (*gfp*) gene (pLW*-fepE*p-*gfp*) and introduced it into strain EDL933-*rfp*, which constitutively expresses the red fluorescent protein (*rfp*) gene from the chromosome. The generated bacteria were intragastrically administered to infant rabbits, and *fepE* promoter activity was measured by quantifying *gfp* expression using quantitative reverse transcription-PCR (qRT-PCR) in EDL933-*rfp* attached to intestinal epithelial cells and unattached bacteria isolated from the intestinal lumen. We used *gfp* as an indirect measurement of *fepE* expression rather than directly measuring *fepE* expression to avoid the interference of *fepE* homologs from intestinal commensal bacteria. *gfp* expression was normalized to *rfp* expression, which did not vary significantly in response to attachment to HeLa cells *in vitro* (Fig. S3). The *gfp* mRNA levels in strain EDL933-*rfp* attached to intestinal epithelial cells were 3.1-fold lower than that in EDL933-*rfp* in the intestinal lumen (Fig. 2E). This finding indicates that *fepE* expression is repressed when strain EDL933 is attached to intestinal epithelial cells *in vivo*, which would result in a reduction in the OAg chain length.

10.1128/mBio.02692-21.3 FIG S3 Relative expression of *rfp* in EDL933-*rfp* after attachment to HeLa cells. The expression of *rfp* in EDL933-*rfp* exhibited no significant variations in response to attachment to the HeLa cell *in vitro*. Download FIG S3, TIF file, 1.1 MB. Copyright © 2021 Liu et al. 2021 Liu et al. https://creativecommons.org/licenses/by/4.0/ This content is distributed under the terms of the Creative Commons Attribution 4.0 International license.

### Long OAg chains contribute to EHEC survival *in vivo* but inhibit bacterial cell adherence.

**FIG 3:**
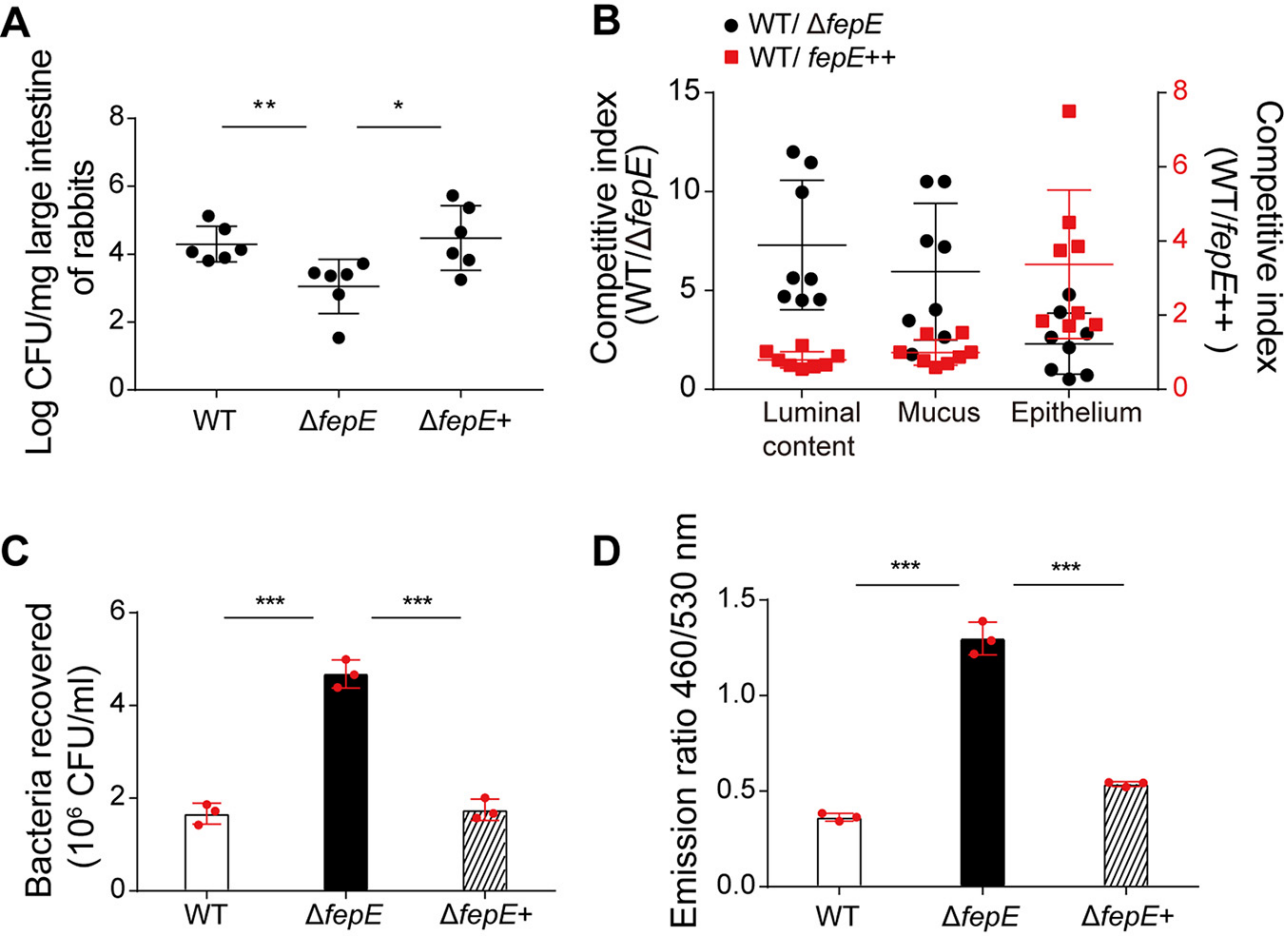
Long OAg chains promote EDL933 colonization *in vivo* but decr]ease adhesion. (A) Determination of the colonization ability of the EDL933 (wild type [WT), EDL933Δ*fepE* (Δ*fepE*), and EDL933Δ*fepE*+ (Δ*fepE*+) strains in the colons of infant rabbits. Bars in the graph represent means ± SD. (B) Competitive index values were calculated as CFU ratio of WT EDL933 versus EDL933Δ*fepE* (WT/Δ*fepE*, black dots, left axis), and the ratio of WT EDL933 versus EDL933-*fepE*++ strain (WT/*fepE*++, red squares, right axis) in the luminal content, mucus, and epithelium of infant rabbits as determined by competitive infection assays. Bars in the graph represent means ± SD. (C) Determination of the adhesion ability of the EDL933 (WT), EDL933Δ*fepE* (Δ*fepE*), and EDL933Δ*fepE*+ (Δ*fepE*+) strains to HeLa cells measured as the HeLa-attached bacteria recovered. (D) Quantification of Map-Bla fusion protein translocation from the EDL933 (WT), EDL933Δ*fepE* (Δ*fepE*) and EDL933Δ*fepE*+ (Δ*fepE*+) strains into HeLa cells. *, *P* < 0.05; **, *P* < 0.01; ***, *P* < 0.001.

*In vivo* infant rabbit colonization experiments using strain EDL933, EDL933Δ*fepE*, and a complemented strain were performed to analyze the effect of OAg chain length on the host colonization efficiency of EDL933. Compared with strain EDL933, the EDL933Δ*fepE* mutant showed significant defects in its colonization ability in the colons of infant rabbits; however, the colonization ability was restored to wild-type levels upon complementation of *fepE* (Fig. 3A), indicating that long OAg chains synthesized in the presence of FepE promote EDL933 colonization *in vivo*.

To investigate whether long OAg chains enhance EDL933 colonization *in vivo* by influencing bacterial survival and/or cell adherence, we performed *in vivo* survival passage experiments in mice using strain EDL933 with an intact *fepE* and with a deleted *fepE*. First, we evaluated the effect of OAg chain length on EDL933 survival in the intestinal lumen by performing the experiments using an EDL933 strain with a Δ*espAD* background, which is unable to adhere to cells due to a nonfunctional T3SS (27). BALB/c mice were administered EDL933Δ*espAD* or EDL933Δ*fepE*Δ*espAD*, and the presence of the bacteria in fecal samples was evaluated 24 h postinoculation. Strain EDL933Δ*espAD* was recovered from 83.3% of fecal samples, a significantly higher percentage than that of strain EDL933Δ*fepE*Δ*espAD* (33.3%) (see Table S1 in the supplemental material), indicating a reduction in the *in vivo* survival ability of EDL933Δ*fepE*Δ*espAD* compared with that of EDL933Δ*espAD*. Complementation of the EDL933Δ*fepE*Δ*espAD* mutant with *fepE* restored its survival ability to a level similar to that of the EDL933Δ*espAD* strain (Table S1). These results indicate that long OAg chains promote EDL933 survival within the intestinal lumen.

10.1128/mBio.02692-21.7 TABLE S1 Recovery of EDL933Δ*espAD*, EDL933Δ*fepE*Δ*espAD*, and EDL933Δ*fepE+*Δ*espAD* from feces after oral administration of the EDL933 mutant strains. Download Table S1, DOCX file, 0.02 MB. Copyright © 2021 Liu et al. 2021 Liu et al. https://creativecommons.org/licenses/by/4.0/ This content is distributed under the terms of the Creative Commons Attribution 4.0 International license.

Next, we evaluated the effect of long OAg chains on EDL933 survival within the colonic lumen and mucus by performing *in vivo* competitive infection assays in infant rabbits using EDL933 and EDL933Δ*fepE* mutant strains and measuring the amount of each bacterium in the intestinal lumen and mucus. A 1:1 mixture of the EDL933 and EDL933Δ*fepE* mutant strains was administered to infant rabbits, and 3 days postinfection, the competitive index (CI) values of the EDL933 strain versus the EDL933Δ*fepE* mutant strain were 7.3 and 6.0 in the luminal content and mucus, respectively (Fig. 3B). These findings indicate that long OAg chains benefit EDL933 survival in the mucus in addition to in the intestinal lumen.

In the intestinal lumen and mucus, antimicrobial peptides are major innate immune factors against pathogens (3, 4). We compared the survival ability of EDL933 and EDL933Δ*fepE* mutant in LB medium with polymyxin B, a representative antimicrobial peptide. The EDL933Δ*fepE* strain exhibited lower survival compared with strain EDL933 when incubated with polymyxin B, whereas complementation with *fepE* restored the survival ability to the wild-type level (Fig. S4). These results indicate that long OAg chains increase the resistance of EDL933 to antimicrobial peptides, which would benefit the survival of EDL933 in the intestinal lumen and mucus.

10.1128/mBio.02692-21.4 FIG S4 Sensitivity of the EDL933 (WT), EDL933Δ*fepE* (Δ*fepE*), and EDL933Δ*fepE*+ (Δ*fepE*+) strains to polymyxin B. Bacteria were incubated with polymyxin B (3 μg/ml) for 1 h. The number of surviving bacteria was determined by plating on solid media. Assays were performed in triplicate. The results are expressed as survival percentages. **, *P* < 0.01. WT denotes wild-type EDL933. Download FIG S4, TIF file, 0.7 MB. Copyright © 2021 Liu et al. 2021 Liu et al. https://creativecommons.org/licenses/by/4.0/ This content is distributed under the terms of the Creative Commons Attribution 4.0 International license.

We then examined the effects of long OAg chains on cell adherence using *in vitro* assays. We found that compared with strain EDL933, the EDL933Δ*fepE* mutant strain exhibited a 2.8-fold increase in the HeLa cell attachment ability, whereas complementation with *fepE* reduced attachment to a level comparable to that of the EDL933 (Fig. 3C). *In vivo* competitive infection assays in infant rabbits were performed with the EDL933 and EDL933-*fepE*++ strain, which overexpresses *fepE*. The CI values of the EDL933 strain versus the EDL933-*fepE*++ strain were 0.8, 1.0, and 3.4 in the luminal content, mucus, and epithelium, respectively, of infant rabbits (Fig. 3B). These findings indicate that long OAg chains inhibit the ability of EDL933 to attach to epithelial cells.

Overall, our results suggest that long OAg chains promote the colonization of EDL933 *in vivo* by increasing its survival; however, long OAg chains inhibit EDL933 attachment to host cells.

### Short OAg chains increase the translocation of bacterial T3SS effector.

Our results so far have showed that strain EDL933Δ*fepE*, which presents mostly short OAg chains, has an increased host cell attachment ability *in vitro* and *in vivo* compared to strain EDL933. Since the translocation of T3SS effectors is required for cell attachment, we next evaluated whether short OAg chains enhance EDL933 cell adhesion by influencing the translocation of T3SS effectors. To this end, we infected HeLa cells with EDL933Δ*fepE* and EDL933 strains transformed with the plasmid pLW*-map*-Bla, in which the *map* gene encoding a T3SS effector (28) was transcriptionally fused to Bla (encoding β-lactamase). We then added the β-lactamase substrate CCF2/AM to the culture and quantified Map-Bla translocation by calculating the shift of green to blue fluorescence occurring following Map-Bla-mediated catalytic cleavage of the CCF2 β-lactam ring (28). The EDL933Δ*fepE* mutant exhibited a 3.6-fold increase in the Map-Bla translocation rate compared with that of the EDL933 strain, and complementation restored the wild-type levels (Fig. 3D). This result indicates that short OAg chains increase the translocation of T3SS effectors.

### Host cell attachment upregulates *hns* expression, which downregulates *fepE* expression, resulting in reduced OAg chain length.

**FIG 4:**
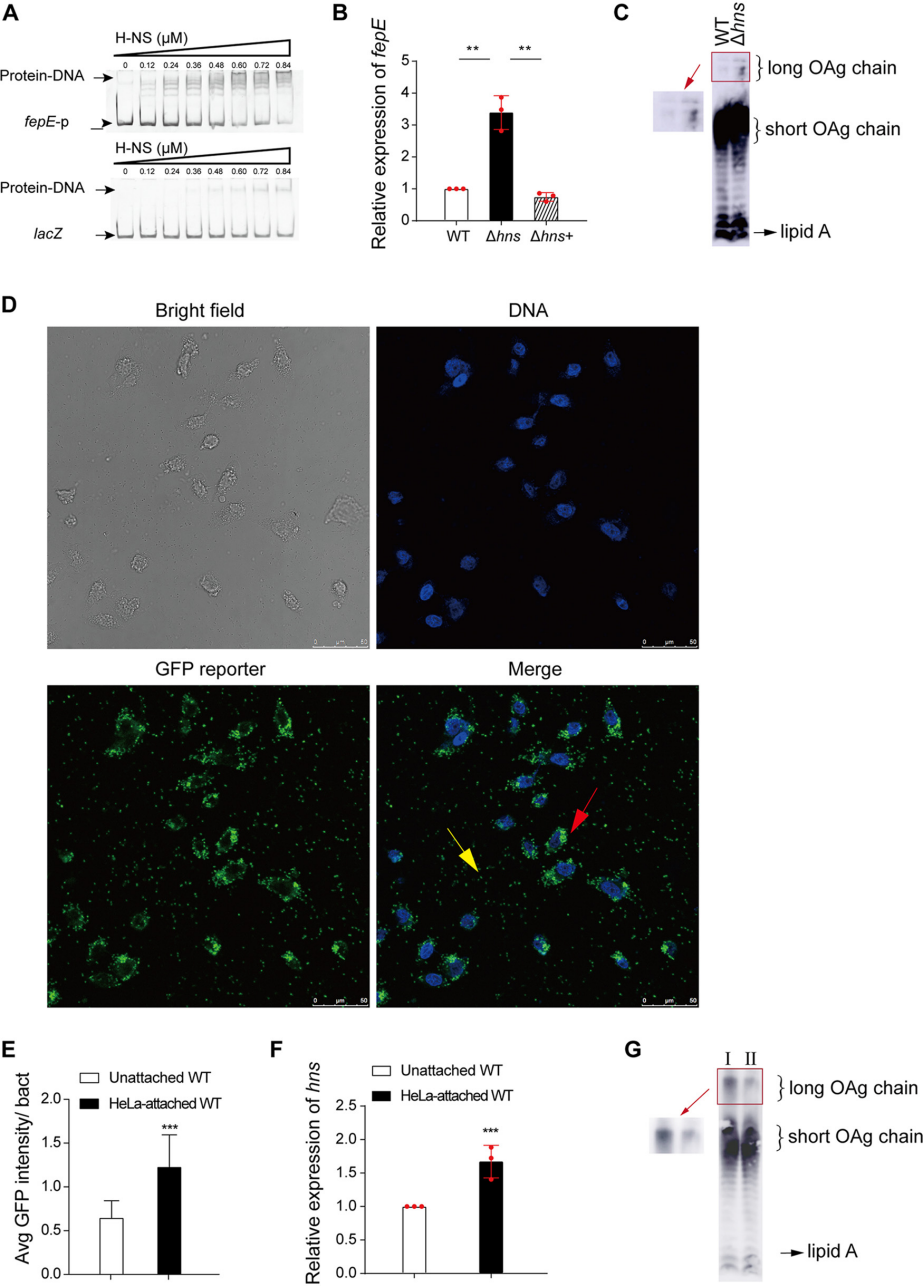
Attachment of strain EDL933 to HeLa cells induces *hns* expression and leads to *fepE* downregulation. (A) EMSA of the *fepE* promoter DNA fragment with purified H-NS−His6 protein. The *lacZ* gene was used as the negative control. (B) Relative expression of *fepE* in EDL933 (WT), EDL933Δ*hns* (Δ*hns*), and EDL933Δ*hns*+ (Δ*hns*+) strains as determined by qRT-PCR. The error bars represent SD. Each experiment was replicated three times independently. (C) Western blotting of LPS obtained from the EDL933 (WT) and EDL933Δ*hns* (Δ*hns*) strains using anti-O157 monoclonal antiserum. (D and E) Confocal fluorescence microscopy analysis of GFP fluorescence intensity produced by HeLa-attached and unattached EDL933 cells containing pLW*-hns*p-*gfp*. The arrows indicate the HeLa-attached (red) and unattached (yellow) EDL933 cells. The data are representative of three independent experiments (>50 bacterial cells per experiment). (F) Relative expression of *hns* in HeLa-attached and unattached EDL933 (WT) cells as determined by qRT-PCR. The error bars represent SD. Each experiment was replicated three times independently. (G) Western blotting of LPS obtained from HeLa-attached EDL933Δ*hns* (I) and unattached EDL933Δ*hns* cells (II) using anti-O157 monoclonal antiserum. **, *P* < 0.01; ***, *P* < 0.001.

Next, we aimed to identify the molecular pathway involved in the regulation of *fepE* expression in response to host cell attachment. We performed DNA pulldown assays to investigate factors that can bind to the promoter region of *fepE* and identified a total of 11 proteins (Fig. S5A and Table S2). One of the identified proteins was H-NS, a well-known global regulator in bacteria (29). The electrophoretic mobility shift assay (EMSA) results showed that H-NS specifically bound to the *fepE* promoter (Fig. 4A). Furthermore, qRT-PCR results showed that *fepE* expression levels were 3.3-fold higher in an EDL933Δ*hns* mutant strain than in the EDL933 strain, and the *fepE* expression levels were restored to wild-type levels after complementation of the EDL933Δ*hns* mutant strain with *hns* (Fig. 4B). To confirm that these changes in *fepE* expression result in changes in the OAg length, we analyzed the production of short and long OAg chains on the EDL933Δ*hns* mutant strain using SDS-PAGE and Western blotting and found that the production of long OAg chains was higher than that on strain EDL933 (Fig. 4C). These findings indicate that H-NS acts as an *fepE* repressor, indirectly affecting OAg chain length.

10.1128/mBio.02692-21.5 FIG S5 Regulation of *fepE* expression and OAg chain length in strain EDL933 during host cell attachment is inhibited when *hns* is deleted. (A) DNA pulldown assay of potential factors binding to the promoter region of *fepE* in EDL933. The proteins were separated by SDS-PAGE, and proteins identified on the gel are listed in Table S2 in the supplemental material. (B) Relative expression of *fepE* in HeLa-attached and unattached EDL933Δ*hns* (Δ*hns*) cells as determined by qRT-PCR. The error bars represent SD. Each experiment was replicated three times independently. (C and D) Surface topology of unattached and HeLa-attached EDL933Δ*hns* (Δ*hns*) cells, as visualized by TEM. The electron-dense material at the bacterial surface is demarcated by the two opposing arrows. In the box plots, center lines show the medians and box lines indicate the 25th and 75th percentiles as determined by R software. The whiskers extend 1.5 times the interquartile range from the 25th and 75th percentiles, with outliers represented by dots. Crosses represent data means, and gray bars indicate 95% confidence intervals of the means. Notches in the boxes emanating from the median values represent the 95% confidence interval for the median values. When the notches of different boxes overlap, the medians are considered not significantly different. The average widths of material extending from the outer membrane in unattached and HeLa-attached EDL933Δ*hns* (Δ*hns*) cells were 23.6 nm and 23.8 nm, respectively. The data are representative of 10 cells from three independent experiments. The regions within the boxes represent magnified views. (E) A major binding site for H-NS in the *fepE* promoter is shown. Electropherogram showing the protection pattern of the *fepE* promoter region after incubation in the presence (red) or absence (blue) of H-NS and subsequent digestion with DNase I. Download FIG S5, TIF file, 2.3 MB. Copyright © 2021 Liu et al. 2021 Liu et al. https://creativecommons.org/licenses/by/4.0/ This content is distributed under the terms of the Creative Commons Attribution 4.0 International license.

10.1128/mBio.02692-21.8 TABLE S2 Proteins identified from the DNA pulldown assay. Download Table S2, DOCX file, 0.02 MB. Copyright © 2021 Liu et al. 2021 Liu et al. https://creativecommons.org/licenses/by/4.0/ This content is distributed under the terms of the Creative Commons Attribution 4.0 International license.

To examine whether *hns* expression is induced during bacterial attachment to HeLa cells, we measured *hns* expression in attached and unattached EDL933 cells containing a plasmid with the *hns* promoter transcriptionally fused to *gfp* (pLW*-hns*p-*gfp*) by detecting green fluorescent protein (GFP) fluorescence by confocal fluorescence microscopy. The average GFP intensity of HeLa-attached bacterial microcolonies was 1.8-fold higher than that of unattached bacterial microcolonies (Fig. 4D and E). Furthermore, qRT-PCR demonstrated that *hns* expression was 1.7-fold higher in HeLa-attached EDL933 cells than in unattached bacterial cells (Fig. 4F). These findings indicate that *hns* expression is upregulated in EDL933 attached to host cells.

We next investigated whether the regulation of *fepE* expression following host cell attachment and the consequent change in OAg chain length are dependent on H-NS. In contrast to that in strain EDL933, *fepE* expression in the EDL933Δ*hns* mutant did not decrease after attachment to HeLa cells, as evidenced by qRT-PCR (Fig. S5B). Furthermore, TEM observation demonstrated that the width of the material extending from the outer membrane of HeLa-attached EDL933Δ*hns* mutant cells was not significantly different from that in unattached EDL933Δ*hns* mutant cells (Fig. S5C and S5D). Consistent with this finding, the SDS-PAGE and Western blotting results showed that the production of long OAg chains on HeLa-attached EDL933Δ*hns* mutant cells was not decreased compared to that on unattached EDL933Δ*hns* mutant cells (Fig. 4G). These results indicate that the upregulation of *fepE* and decrease of OAg chain length in strain EDL933 in response to host cell attachment are mediated by H-NS.

Collectively, these results indicate that upon EDL933 attachment to host cells, *hns* is upregulated, causing *fepE* downregulation and leading to a reduction in the OAg chain length.

### The membrane protein YgjI senses host cell attachment and regulates *fepE* expression via H-NS.

To investigate whether a specific bacterial envelope component is involved in sensing host cell attachment signals and transducing these signals to H-NS in the cytoplasm of EDL933 cells, we constructed global mutant libraries of EDL933 strains harboring plasmids containing the *hns* promoter transcriptionally fused to the *lux* reporter gene (pLW*-hns*p-*lux*). The mutant libraries (30,000 mutants) were screened *in vitro* to identify regulators of *hns* by analyzing cell luminescence. The luminescence levels of nine gene mutants (Table S3) were significantly altered (>2-fold) compared with those of the EDL933 strain harboring pLW*-hns*p-*lux*, indicating that the corresponding mutated genes possibly influence *hns* expression. We then assessed whether these potential *hns* regulators regulated *hns* expression in strain EDL933 attached to HeLa cells. To this end, we constructed the mutants for these nine genes in EDL933 and introduced pLW*-hns*p-*gfp* into each of the nine mutants. Next, we measured *hns* expression of these generated strains following attachment to HeLa cells by detecting GFP fluorescence with confocal fluorescence microscopy. The GFP fluorescence intensities of eight HeLa-attached mutants were higher than those of the corresponding unattached bacteria and were similar to those of the EDL933 strain, indicating that mutation of these eight genes did not inhibit *hns* upregulation in response to HeLa cell attachment signals. In contrast, the EDL933Δ*ygjI* mutant did not exhibit increased GFP fluorescence when attached to HeLa cells (Fig. S6), indicating that *hns* expression is not upregulated in this mutant after HeLa cell attachment.

10.1128/mBio.02692-21.6 FIG S6 EDL933Δ*ygjI* mutant containing pLW*-hns*p-*gfp* did not exhibit increased GFP fluorescence intensity following attachment to HeLa cells. (A) Confocal fluorescence microscopy was performed to assess GFP expression in HeLa-attached and unattached Δ*ygjI* cells containing pLW*-hns*p-*gfp*. The arrows indicate bacteria attached (red) or unattached (yellow) to HeLa cells. The data are representative of three independent experiments (>50 bacterial cells per experiment). (B) Fluorescence intensities of GFP in unattached and HeLa-attached EDL933Δ*ygj.* Download FIG S6, TIF file, 2.3 MB. Copyright © 2021 Liu et al. 2021 Liu et al. https://creativecommons.org/licenses/by/4.0/ This content is distributed under the terms of the Creative Commons Attribution 4.0 International license.

10.1128/mBio.02692-21.9 TABLE S3 Gene mutants containing pLW-*hn*sp-*lux*, whose luminescence intensities were significantly altered compared with those of the EDL933 strain harboring pLW-*hns*p-*lux*. Download Table S3, DOCX file, 0.02 MB. Copyright © 2021 Liu et al. 2021 Liu et al. https://creativecommons.org/licenses/by/4.0/ This content is distributed under the terms of the Creative Commons Attribution 4.0 International license.

**FIG 5:**
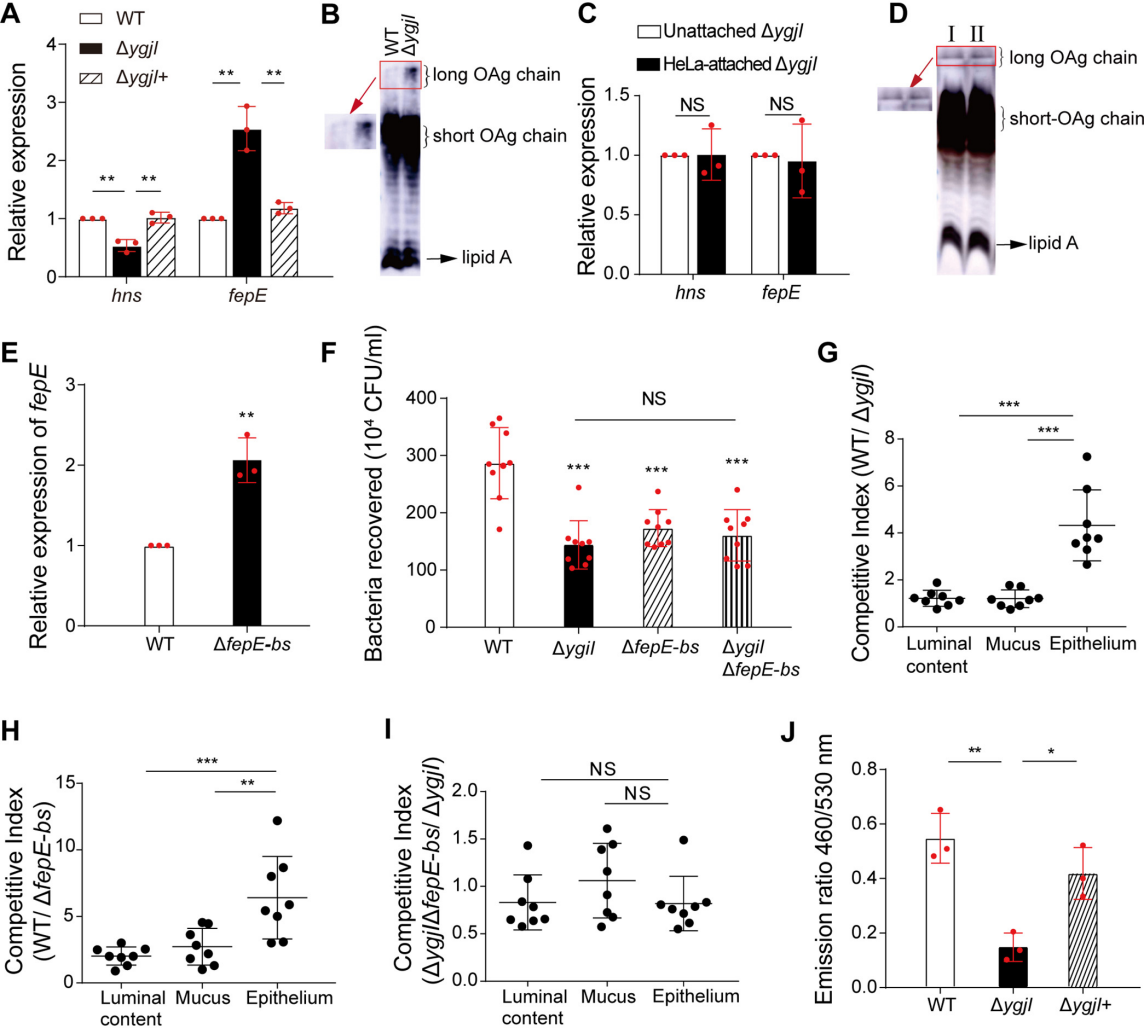
YgjI is involved in the sensing of host cell attachment signals by strain EDL933 and upregulates *hns* expression. (A) Relative expression of *fepE* and *hns* in the EDL933 (WT), EDL933Δ*ygjI* (Δ*ygjI*), and EDL933Δ*ygjI*+ (Δ*ygjI*+) strains as determined by qRT-PCR. The error bars represent SD. Each experiment was replicated three times independently. (B) Western blotting of LPS obtained from the EDL933 (WT) and EDL933Δ*ygjI* (Δ*ygjI*) strains using anti-O157 monoclonal antiserum. (C) Relative expression of *fepE* and *hns* in HeLa-attached and unattached EDL933Δ*ygjI* (Δ*ygjI*) cells as determined by qRT-PCR. The error bars represent SD. Each experiment was replicated three times independently. (D) Western blotting of LPS prepared from HeLa-attached EDL933Δ*ygjI* (I) and unattached EDL933Δ*ygjI* (II) cells using anti-O157 monoclonal antiserum. (E) Relative expression of *fepE* in the EDL933 (WT) and EDL933Δ*fepE-bs* (Δ*fepE-bs*) strains, as determined by qRT-PCR. The error bars represent SD. Each experiment was replicated three times independently. (F) Determination of the adhesion ability of the EDL933 (WT), EDL933Δ*ygjI* (Δ*ygjI*), EDL933Δ*fepE-bs* (Δ*fepE-bs*), and EDL933Δ*ygjI*Δ*fepE-bs* (Δ*ygjI*Δ*fepE-bs*) strains to HeLa cells determined as the number of the HeLa-attached bacteria recovered. (G to I) Competitive index values were calculated as CFU ratio of EDL933 versus EDL933Δ*ygjI* (WT/Δ*ygjI*) (G), EDL933 versus EDL933Δ*fepE-bs* (WT/Δ*fepE-bs*) (H), and EDL933Δ*ygjI*Δ*fepE-bs* versus EDL933Δ*ygjI* (Δ*ygjI*Δ*fepE-bs*/Δ*ygjI*) (I) in the luminal content, mucus, and epithelium of infant rabbits as determined by competitive infection assays. (J) Quantification of Map-Bla fusion protein translocation from the EDL933(WT), EDL933Δ*ygjI*(Δ*ygjI*+), and EDL933Δ*ygjI*+(Δ*ygjI*+) strains into HeLa cells. *, *P* < 0.05; **, *P* < 0.01; ***, *P* < 0.001; NS, not significant.

YgjI is an inner membrane protein with 12 potential transmembrane segments (30) and contains an amino acid permease motif (pfam13520), yet its function remains unknown. The qRT-PCR results showed that the *hns* and *fepE* expression levels in unattached EDL933Δ*ygjI* mutant decreased by 1.9-fold and increased by 2.5-fold, respectively, compared with those in the EDL933 strain, and that the wild-type expression levels were restored after complementation with *ygjI* (Fig. 5A). Consistent with these findings, the results of SDS-PAGE and Western blotting showed that the production of long OAg chains on the EDL933Δ*ygjI* mutant was increased compared to that on the EDL933 strain (Fig. 5B). We next investigated whether YgjI is required for *hns* upregulation, *fepE* downregulation, and OAg chain length reduction in response to host cell attachment. The qRT-PCR results indicated that expression of *hns* and *fepE* was not induced or repressed, respectively, in EDL933Δ*ygjI* mutant cells upon attachment to HeLa cells (Fig. 5C). Finally, SDS-PAGE and Western blotting showed that the production of long OAg chains on HeLa-attached EDL933Δ*ygjI* mutant cells was not decreased compared to that on unattached EDL933Δ*ygjI* mutant cells (Fig. 5D). These findings indicate that YgjI is involved in sensing host cell attachment signals and that it induces *hns* expression, which regulates *fepE* expression, thereby reducing the OAg chain length during EDL933 attachment to host cells.

We next investigated whether YgjI contributes to the attachment of strain EDL933 to epithelial cells by regulating *fepE* expression via H-HS. H-NS binds to AT-rich regions in the promoters of the genes it regulates (29). We identified a major H-NS-bound sequence (5′-TTTGCTGGTTATT-3′) in the *fepE* promoter region using a dye-based DNase I footprinting assay (Fig. S5E), and this sequence was deleted to create the mutant strain EDL933Δ*fepE-bs*. The results of qRT-PCR assays showed that *fepE* expression in the unattached EDL933Δ*fepE-bs* mutant exhibited a 2.1-fold increase compared to that in the unattached EDL933 strain (Fig. 5E), indicating the regulation of H-NS on *fepE* expression is inhibited in this mutant. We also constructed EDL933Δ*ygjI*Δ*fepE-bs* mutants, as we failed to obtain the EDL933Δ*ygjI*Δ*fepE* mutant. Cell adherence assays showed that the EDL933Δ*ygjI* and EDL933Δ*fepE-bs* mutants exhibited decreases of 2.0-fold and 1.7-fold, respectively, in their HeLa cell attachment ability compared with that of the EDL933 strain (Fig. 5F). However, the cell attachment ability of the EDL933Δ*ygjI*Δ*fepE-bs* mutant did not decrease more than that of the EDL933Δ*ygjI* mutant (Fig. 5F). *In vivo* competitive infection assays in infant rabbits were performed between the EDL933 strain and the EDL933Δ*ygjI* mutant as well as between the EDL933 strain and the EDL933Δ*fepE-bs* mutant, and the CI values were calculated in the intestinal lumen, mucus, and epithelium. We found that the ability of both mutants to attach to epithelial cells was significantly decreased compared with that of the EDL933 strain, but the survival ability of these strains was similar to that of the EDL933 strain within the lumen and mucus of the colon (Fig. 5G and H). Furthermore, *in vivo* competitive infection assays between the EDL933Δ*ygjI*Δ*fepE-bs* mutant and the EDL933Δ*ygjI* mutant showed CI values of 0.8, 1.0, and 0.8 in the intestinal lumen, mucus, and epithelium, respectively (Fig. 5I), indicating that the EDL933Δ*ygjI* and EDL933Δ*ygjI*Δ*fepE-bs* mutants were not significantly different in their ability to attach to epithelial cells. These data indicate that YgjI contributes to bacterial attachment to epithelial cells via *fepE*.

The above results show that short OAg chains promote the translocation of T3SS effectors, and YgjI contributes to OAg chain length reduction in response to host cell attachment. Thus, we next investigated whether YgjI expression affects T3SS function. We quantified the translocation of T3SS effectors from the EDL933Δ*ygjI* and EDL933 strains into HeLa cells using the Map-Bla fusion protein system described above (28). The translocation rate of the Map-TEM fusion protein in the EDL933Δ*ygjI* strain was decreased by 4.2-fold compared with that of the EDL933 strain, and complementation with *ygjI* restored the wild-type levels (Fig. 5J). These results indicate that YgjI promotes the translocation of T3SS effectors, which ultimately enhances the bacterial infectivity of intestinal epithelial cells.

### Activation of the YgjI−H-NS−FepE regulatory pathway is induced by a mechanical signal following host cell attachment.

**FIG 6:**
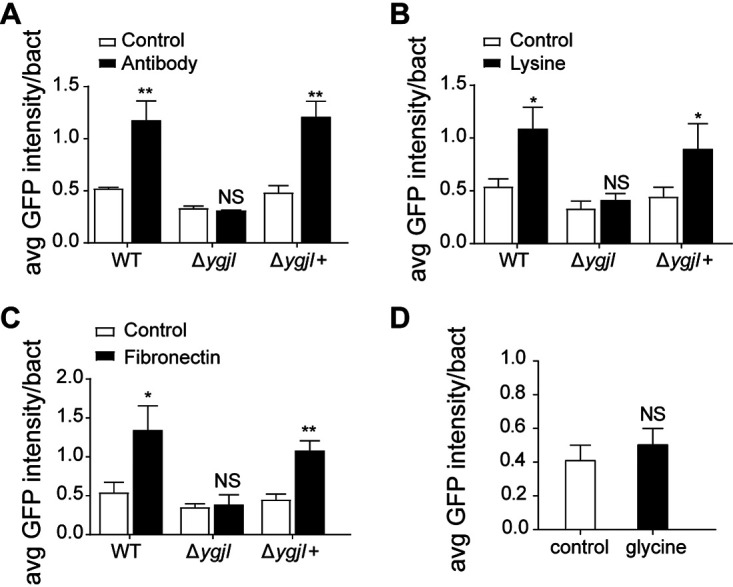
Strain EDL933 senses host cell attachment as a mechanical cue to regulate OAg chain length. (A to C) GFP fluorescence intensity of EDL933 (WT), EDL933Δ*ygjI* (Δ*ygjI*), and EDL933Δ*ygjI*+ (Δ*ygjI*+) cells containing pLW*-hns*p-*gfp* immobilized on coverslips coated with antiserum against O157 antigen (A), poly-l-lysine (B), or fibronectin (C) or on uncoated coverslips (control). (D) GFP fluorescence intensity of EDL933 cells harboring pLW*-hns*p-*gfp* immobilized on coverslips coated with glycine. *, *P* < 0.05; **, *P* < 0.01; NS, not significant.

Finally, we investigated the host cell attachment signal that activates the YgjI−H-NS−FepE pathway resulting in OAg chain length reduction and enhanced host cell adhesion. Specifically, we investigated whether this signal was a mechanical cue or a specific chemical feature or receptor on the host cell surface. EDL933 and EDL933Δ*ygjI* mutant cells containing the reporter plasmid pLW*-hns*p-*gfp* were immobilized on coverslips coated with three different substrates mimicking the three types of bacterium-host cell interactions: positively charged poly-l-lysine (that binds to the negatively charged bacterial cell wall), antisera against O157 antigens, and fibronectin, which is the receptor for several adhesion factors on the surfaces of host cells (31). Immobilization of strain EDL933 containing pLW*-hns*p-*gfp* on all these substrates induced an increase in the GFP fluorescence intensity compared with immobilization of bacterial cells on uncoated control coverslips (Fig. 6A, B, and C). In contrast, no increase was observed in the GFP fluorescence intensity of EDL933Δ*ygjI* mutant containing pLW*-hns*p-*gfp* immobilized on coated coverslips compared with that of the same bacteria immobilized on uncoated control coverslips; this lack of induction was reversed in the *ygjI* complemented strain (Fig. 6A, B, and C). For a control, we measured GFP fluorescence intensity in strain EDL933 containing pLW*-hns*p-*gfp* immobilized on coverslips coated with negatively charged glycine and found no increase in the GFP fluorescence intensity compared to that on uncoated coverslips (Fig. 6D). These findings suggest that *hns* upregulation mediated by YgjI is induced in response to a mechanical signal generated by host cell attachment and is independent of the type of interaction between the bacterial cell and host cell surface.

## DISCUSSION

The goals of this study were to investigate the regulation of EHEC OAg chain length in the intestinal lumen, mucus, and epithelium, explore the underlying mechanisms, and analyze the influence of this regulation on bacterial survival and pathogenesis *in vivo*. We reveal a novel mechanism by which EHEC regulates its OAg chain length via a membrane protein YgjI during the infection process. In the intestinal lumen and mucus, EHEC O157 expresses long-chain OAg that promotes its survival. When EHEC O157 establishes initial attachment to intestinal epithelial cells, bacteria sense the mechanical signal of this attachment via YgjI, which upregulates *hns* expression. In turn, H-NS upregulation directly represses *fepE* expression, leading to a reduction in the OAg chain length. The short OAg chains enhance EHEC infectivity by promoting intimate attachment of bacteria by increasing the injection of T3SS effectors into epithelial cells. This regulatory pathway is important for the virulence of EHEC O157, as it not only promotes bacterial infection of intestinal epithelial cells but also enhances bacterial survival in the intestinal lumen.

During the infection process, EHEC must first survive components of the host defense systems, such as gastric acid and different innate immune factors. Mammalian intestinal epithelial cells secrete antimicrobial peptides into the mucus and intestinal lumen. Bacteria have developed several strategies that resist the actions of antimicrobial peptides, such as modification of lipid A that decreases the negative charge of the bacterial membrane, proteolytic degradation of peptide, and export of peptides by efflux pumps (6). It was also reported that a Salmonella mutant with short-chain OAg exhibits decreased resistance to antimicrobial peptides *in vitro* (32). Our work demonstrated that bacteria express long-chain OAg in host intestinal lumen and provided evidence that long OAg chains increase the resistance of bacteria to antimicrobial peptides, enhancing bacterial survival in the intestinal lumen and mucus.

Our work shows that attachment of EHEC O157 to intestinal epithelial cells reduces OAg chain length, leading to enhanced translocation of T3SS effectors. The T3SS of EHEC contains a filamentous needle, in which a filamentous structure extends the needle toward the host cell (11). Upon contact between the filamentous needle and the host cell, a translocation pore is formed in the host cell membrane that initiates effector translocation (33). As OAg is the major constituent extending outward from the bacterial surface and T3SS expression is increased in host cell-attached EHEC cells (34), it is highly likely that the reduction in the OAg chain length during EHEC attachment enhances the access of the newly assembled T3SS to the host cell surface, contributing to increased T3SS effector translocation. Furthermore, it is possible that the *ygjI*-*hns*-*fepE* regulatory pathway is under the control of positive-feedback regulation. Attachment of EHEC to host cells mediated by T3SS-secreted effectors, such as intimin (23), may induce the YgjI−H-NS−FepE pathway, reinforcing the existing attachment of EHEC to host cells.

In this study, we find that EHEC O157 expresses long-chain OAg in the intestinal lumen and shortens its OAg chain length when attached to intestinal epithelial cells. EHEC has been shown to activate virulence gene expression upon sensing several intestinal chemical signals (35–38). However, chemical signals are unlikely to regulate the OAg chain length in the intestinal lumen and epithelium. This is because the intestinal epithelium directly surrounds the intestinal lumen, and differences in the concentration of chemical signals between these regions are likely extremely limited. Thus, bacteria cannot precisely regulate OAg chain length in the intestinal lumen and epithelium by sensing chemical signals. Therefore, detecting mechanical signals of host cell attachment is a more precise and reliable mechanism by which EHEC initiates the regulation of its OAg chain length.

The abilities of several bacterial pathogens to sense and respond to mechanical signals generated from surface attachment have been recently discovered, mainly by investigating bacterial phenotypes when attaching to a variety of distinct abiotic surfaces and host cells (34, 39–42). In this study, we showed that attachment of strain EDL933 to host cells or coverslips coated with three different substrates (positively charged poly-l-lysine, O157 antiserum, and fibronectin) activated the YgjI−H-NS−FepE pathway. The findings indicate that the activation of this regulatory pathway occurs in response to a mechanical signal generated by host cell attachment. Nonetheless, future studies are necessary to confirm this finding. For instance, whether the immobilization of strain EDL933 on other receptors (e.g., Tir) present on the surfaces of host cells also induce the YgjI−H-NS−FepE pathway is needed to be assessed. In addition, experiments with microfluidic flow system can be performed to measure whether the force acting perpendicular to the cell wall, which is similar to the force generated by host cell attachment, is able to activate this pathway. It is also worth investigating whether fluid shear force present in the host intestinal tract, which has been previously reported as a mechanical signal that can be sensed by bacteria (34, 40, 42), can further activate the YgjI−H-NS−FepE pathway in attached bacteria.

Numerous Gram-negative bacteria, including E. coli, constitutively produce outer membrane vesicles (OMVs), which can remove a portion of the outer membrane containing LPS, resulting in newly synthesized LPS molecules replacing “old” LPS molecules constantly (43–46). In this study, we showed that upon sensing host cell attachment signals, EHEC cells start to synthesize short-chain OAg by repressing *fepE* expression. As EHEC cells remove “old” OAg (including long-chain OAg) via OMVs all the time, this process may eventually enable attached EHEC cells to replace long-chain OAg with short-chain OAg in their outer membrane. Moreover, it is also possible that EHEC still grows and replicates when attached to epithelial cells and can reduce the chain length of newly synthesized OAg during this process by repressing *fepE* expression.

Very few reports have addressed the mechanisms by which bacteria regulate gene expression or translation upon sensing host cell attachment signals. In the few cases reported, bacteria sense these signals via a factor in their outer membrane. For instance, enteropathogenic E. coli (EPEC) senses host cell attachment via T3SS, which triggers the production of the effector NleA (47), while Pseudomonas aeruginosa transduces surface attachment signals via type IV pili and the outer membrane protein PilY, leading to induction of virulence gene expression (40, 41). Therefore, in addition to the inner membrane protein YgjI, EHEC possibly directly senses cues related to mechanical attachment to epithelial cells via an outer membrane factor, thus initiating the regulation of the OAg chain length. The mechanical signal would be transduced to YgjI likely through the periplasmic domain of this outer membrane factor or via another periplasmic protein. Furthermore, YgjI does not contain a cytoplasmic domain; thus, we hypothesize that there is another inner membrane protein with a cytoplasmic domain and regulatory function linking the signal transduction between YgjI and H-NS. Further research is required to elucidate this signal transduction pathway.

We found that the genes *ygjI*, *hns*, and *fepE* are present in all 576 available complete E. coli genomes. We also analyzed *fepE* expression in three other EHEC strains (O26:H11, O111:H8, and O145:H28) and three EPEC strains (O55:H7, O114:H2, and O127:H7) in response to host cell attachment. The results showed that for all these six strains, *fepE* expression was significantly repressed following attachment to HeLa cells or Caco-2 cells (Fig. S2). These data indicate that the YgjI−H-NS−FepE pathway regulating the OAg chain length may also be present in other pathogenic E. coli strains and contribute to their virulence. Although some pathogenic E. coli strains, such as enterotoxigenic E. coli (ETEC) and enteroaggregative E. coli (EAEC), do not express a T3SS, a short OAg chain length may contribute to efficient interactions between the surfaces of bacterial cells and host cells. This pathway could be a promising target to control the intestinal infections caused by pathogenic E. coli.

## MATERIALS AND METHODS

### Bacterial strains and plasmids.

The bacterial strains and plasmids used in this study are summarized in Table S4 in the supplemental material. Mutant strains were generated using the λ Red recombinase system (48), and all strains were verified via PCR amplification and sequencing. All primers used in this study are listed in Table S4. Genes of interest together with their native promoters were cloned into the pACYC184 plasmid, and the recombinant plasmid was then electroporated into the corresponding mutant strain to construct the complemented strain. The EDL933-*fepE*++ strain was constructed via introducing a recombinant pTRC99a plasmid containing the *fepE* gene fused with a constitutive promoter, BBa_J23119, into strain EDL933 (49).

10.1128/mBio.02692-21.10 TABLE S4 Strains, plasmids, and primers used in this study. Download Table S4, DOCX file, 0.03 MB. Copyright © 2021 Liu et al. 2021 Liu et al. https://creativecommons.org/licenses/by/4.0/ This content is distributed under the terms of the Creative Commons Attribution 4.0 International license.

### Bacterial adherence assay.

HeLa cells were purchased from the Shanghai Institute of Biochemistry and Cell Biology of the Chinese Academy of Sciences (Shanghai, China). In brief, HeLa cells were trypsinized, resuspended, and seeded in 90-mm cell culture plates (NEST) at the concentration of 5 × 10^6^ cells per plate 1 day before infection. Cells were grown in Dulbecco modified Eagle medium (DMEM) (HyClone, Logan, UT, USA) supplemented with 10% fetal bovine serum (ExCell, Shanghai, China) and incubated at 37°C in an atmosphere containing 5% CO_2_. The same batch of HeLa cells was simultaneously used in the control and experimental groups to avoid significant differences in cell numbers. During infection, cell monolayers were challenged with bacteria grown in liquid culture (5 × 10^8^ CFU) at a multiplicity of infection of 100 and then incubated for 3 h at 37°C in an atmosphere containing 5% CO_2_. After incubation, unattached bacteria were collected from the supernatant and resuspended in 10 ml of precooled sterile phosphate-buffered saline (PBS) to remove the residual DMEM. The supernatant was discarded after centrifuging at 5,500 rpm at 4°C for 3 min. The pellet of unattached bacteria was washed six times with PBS. The host cells were washed as previously described (50). Briefly, 2 ml of precooled sterile PBS was added to the culture plate and wobbled back and forth in different directions to wash the host cells and then removed. The wash step was repeated six times. Then, 1 ml of 0.1% Triton X-100 was added to the plate to lyse the HeLa cells, and the attached bacteria were collected. The entire wash and collection process for HeLa-attached and unattached bacteria was completed within 20 min. Samples were then used for adhesion ability determination or stored at −80°C for subsequent qRT-PCR. To determine the adhesion ability, 100 μl of the lysate was diluted in gradient with PBS and recovered by plating on LB agar medium in the presence of a particular antibiotic. Adherence was assessed by determining the viable count (CFU per milliliter). The experiment was independently repeated at least three times.

### RNA extraction and qRT-PCR.

To obtain sufficient RNA from the HeLa-attached bacteria, we simultaneously seeded two parallel culture plates (90-mm diameter). The attached bacteria were collected in one centrifuge tube for RNA extraction. This provided sufficient RNA for the processing of cDNA. Sample pellets for RNA extraction were refrigerated in liquid nitrogen and then transferred to a sterile mortar precooled with liquid nitrogen. The pellets were pulverized in liquid nitrogen and lysed with 1 ml TRIzol LS Reagent (Invitrogen, Carlsbad, CA, USA). RNA extraction was performed according to the manufacturer’s instruction. Extracted RNA was treated with RNase-free DNase I (Fermentas, Waltham, MA, USA) to eliminate genomic DNA contamination. cDNA was synthesized using a PrimeScript 1st Strand cDNA synthesis kit (TaKaRa Bio, Shiga, Japan). qRT-PCR analysis was performed on an Applied Biosystems ABI 7300 real-time PCR system (Applied Biosystems, Franklin Lakes, NJ, USA). Each qRT-PCR was carried out in a total volume of 20 μl in a 96-well optical reaction plate (Applied Biosystems) containing 10 μl Power SYBR green PCR Master Mix (Applied Biosystems), 1 μl cDNA, and two gene-specific primers with a final concentration of 0.3 mM each. The *gyrB* gene was used as the reference control for normalization. The relative differences in gene expression were calculated using the 2^−ΔΔCt^ method, which calculated the relative fold gene expression between the experimental sample and the control sample (51). Ct stands for the cycle threshold of the sample generated by qRT-PCR; ΔCt = Ct (the target gene) − Ct (*gyrB*), and ΔΔCt = ΔCt (experimental sample) − ΔCt (control sample). At least three biological replicates were established for each qRT-PCR analysis.

### Extraction and Western blotting of LPS.

LPS was extracted using hot aqueous-phenol extraction. To control the loading quantity of extracted LPS of the target samples, a colorimetric anthrone-sulfuric acid method was used to quantify the polysaccharides. Equal amounts of LPS from each sample were separated by 13% SDS-PAGE and transferred to polyvinylidene difluoride membranes. The membranes were treated with 5% bovine serum albumin for 1 h to block nonspecific binding and incubated with mouse monoclonal E. coli O157 antibody (1:100 dilution; Fitzgerald, Acton, MA, USA) overnight at 4°C, followed by washing in Tris-buffered saline containing Tween (TBST). The blots were further incubated with the secondary antibody goat anti-mouse antibody labeled with horseradish peroxidase (HRP) (1:20,000 dilution, Abcam, Cambridge, UK) for 1 h. Blots were washed in TBST followed by detection using enhanced chemiluminescence (ECL) reagent (Sangon Biotech, Shanghai, China).

### Infant rabbit model of EHEC infection.

Laboratory animals were purchased from Beijing Vital River Laboratory Animal Technology Co. Ltd. (Beijing, China). Infant rabbit experiments were conducted as previously described (52). In brief, 3-day-old New Zealand White rabbits were intragastrically inoculated with 10^8^ CFU of bacteria (EDL933Nal^R^, EDL933Δ*fepE*, and EDL933Δ*fepE*+) in the logarithmic phase of growth. The rabbits were euthanized 3 days postinfection via deep anesthesia with sodium pentobarbital. The colons were excised, and the luminal contents were removed. The colons were weighed, washed, and homogenized in PBS. The homogenates were subsequently diluted and recovered on LB agar containing nalidixic acid (50 μg/ml) to determine the number of bacteria in the colon (CFU per milligram).

### Bacterial competition experiments in rabbits.

Competitive assays in rabbits were performed to compare bacterial survival within the colonic lumen and mucus or compare bacterial attachment to epithelium, between two different strains by measuring the bacterial amount in different intestinal locations (53, 54). Two different strains were mixed in a 1:1 ratio (5 × 10^8^ CFU for each strain). The mixture (100 μl) was intragastrically inoculated into 3-day-old New Zealand White rabbits. The rabbits were euthanized 3 days after infection by deep anesthesia with 3% sodium pentobarbital. The colons were excised and split open with a scalpel. The luminal contents were retrieved with tweezers, and the remaining debris was removed by gentle flushing with PBS. The mucus was gently scraped off the epithelia with a cell scraper as previously described (55, 56). The separated luminal contents, mucus, and epithelium were homogenized in PBS. The homogenates were subsequently diluted and recovered in duplicate on LB agar containing the corresponding antibiotics for selection. The CFU were determined and used to calculate the CI values.

### Mouse survival passage experiments.

Passage analyses were conducted as previously described (57). In brief, mutant strains (EDL933Δ*espAD*, EDL933Δ*fepE*Δ*espAD*, and EDL933Δ*fepE*+Δ*espAD*) were grown to logarithmic phase in LB at 37°C, and 10^4^ CFU of bacteria was then orally administered to individually housed 6-week-old female BALB/c mice (*n* = 4 per group), which were purchased from Beijing Vital River Laboratory Animal Technology Co. Ltd. To allow inoculum clearance through the stomach, the mice were not provided food for 4 h after oral administration. Fecal samples were collected 24 h after inoculation, suspended in PBS (0.5 g feces/4.5 ml PBS) and subsequently diluted. The diluted samples were then plated on LB agar containing kanamycin (25 μg/ml) to evaluate the presence or absence of the corresponding strains. Three independent trials were performed.

### DNA pulldown assay.

The DNA pulldown assay was carried out as previously described (58). In brief, biotinylated DNA was conjugated to M-280 Dynabeads (Invitrogen, Carlsbad, CA, USA) and washed with BS-THES buffer (5× BS buffer: 50 mM HEPES, 25 mM CaCl_2_, 250 mM KCl, 60% glycerol, 1.5% protease inhibitor, 0.2% phosphatase inhibitor; 2.25× THES: 50 mM Tris-HCl [pH 7.5], 10 mM EDTA, 20% sucrose, 140 mM NaCl) containing nonspecific DNA. BS-THES buffer along with cleared bacterial lysate was applied to a DNA-bead complex, with 25 to 100 μg of nonspecific DNA added, and incubated at room temperature for 30 min. The bead-DNA-protein complex was pulled down using a magnetic column (Promega, Madison, WI, USA). These steps were rolled for three times to gain more bead-DNA-protein complex. The complex was washed five times with BS-THES buffer supplemented with nonspecific DNA, followed by two washes with BS-THES buffer. Elution was performed with 100 mM NaCl elution buffer to remove nonadherent and low-specificity DNA-binding proteins. Then, 100 mM NaCl, 200 mM NaCl, 300 mM NaCl, and 500 mM NaCl elution buffers were used to elute higher-specificity DNA-binding proteins. The eluted proteins were then separated by SDS-PAGE and identified by mass spectrometry.

### Electrophoretic mobility shift assay (EMSA).

Gel mobility shift assays were performed by incubating amplified DNA fragments containing the promoter region of *fepE* (1 nM) at 37°C for 30 min with various concentrations of H-NS protein (0 to 200 nM) in 20 μl of a solution containing 20 mM Tris-HCl (pH 7.5), 80 mM NaCl, 0.1 mM EDTA, 1 mM dithiothreitol, and 10% glycerol. Amplified *lacZ* DNA fragments were used as the negative control. Samples were loaded with native binding buffer on 6.0% nondenaturing polyacrylamide gels in 0.5× Tris-borate-EDTA (TBE) buffer. Gels were stained with GelRed and visualized using Tanon 3500 (Tanon, Shanghai, China).

### Dye primer-based DNase I footprinting assay.

The *fepE* promoter was amplified with modified EMSA primers (with a fluorescein amidite [FAM] label at the 5′ end of the forward primer) and incubated with 3.4 μmol/liter H-NS in EMSA buffer containing 0.2 mM CaCl_2_ and 2.5 mM MgCl_2_. The protein-DNA mixtures were partially digested with 0.05 U DNase I for 5 min at 25°C. The reaction was quenched by heating at 75°C for 10 min, and the products were purified using SanPrep Column PCR Product purification kit (Sangon Biotech). Control samples were prepared without H-NS protein. The genotype samples were analyzed by Vebery Biotechnology Co., Ltd. (Shanghai, China).

### Quantification of T3SS translocation.

The recombinant plasmid encoding the Map-Bla fusion protein was constructed and electroporated into the indicated strains to analyze the translocation efficiency of Map-Bla as previously described (28). In brief, HeLa cells were trypsinized and seeded onto black 96-well plates with a clear bottom (Corning, Inc., Corning, NY, USA). Cell monolayers were then challenged with bacteria grown in liquid culture (optical density at 600 nm [OD_600_] of 0.2 to 0.25) at a multiplicity of infection of 100 and incubated at 37°C in an atmosphere containing 5% CO_2_. After 30 min of infection, isopropyl-β-d-1-thiogalactopyranoside (IPTG) was added to induce the production of β-lactamase, and infection was allowed to proceed for an additional hour. The cell monolayers were washed and covered with CCF2/AM solution freshly prepared with a CCF2/AM loading kit (Invitrogen) and were then incubated for 90 min at room temperature. Fluorescence intensity was quantified using a microplate reader (BioTek, Winooski, VT, USA) at an excitation wavelength of 405 nm, and emission was detected through 460-nm (blue fluorescence) and 530-nm (green fluorescence) filters. Translocation was expressed as the 460-nm/530-nm emission ratio in order to normalize β-lactamase activity to cell loading and the number of cells in each well. The data are presented as the means of results from triplicate wells from three experiments.

### Transposon mutagenesis and screening assay.

The pLW-*hns*p-*lux* recombinant plasmid was constructed and electroporated into EDL933 cells. Transposon mutagenesis of pLW-*hnsp*-*lux*-harboring EDL933 using the Tn*5*-RL27 transposon was performed as previously described (59). In brief, chloramphenicol and kanamycin were used for selection after delivery of the conjugal plasmid bearing Tn*5*-RL27. After overnight incubation at 37°C, monoclones were cultured in 96-well plates, and luminescence was then measured with a microplate reader. A total of 30,000 mutants were screened, and mutants exhibiting significantly altered luminescence levels compared with those of the wild-type strain harboring pLW-*hns*p-*lux* were selected for further identification.

### Surface coating with pure substrates to assess bacterial adhesion.

This experiment was performed as previously described (34). In brief, surfaces were coated with poly-l-lysine (Beyotime, Shanghai, China), glycine (Invitrogen), O157 antiserum (Fitzgerald), or fibronectin (Thermo Fisher Scientific, Waltham, MA, USA), and strains containing the pLW-*hns*p-*gfp* plasmid were then immobilized on the substrate-coated coverslips and incubated for 2 h under static conditions. Next, the average fluorescence intensity of individual cells was determined by image analysis. The data are representative of three independent experiments.

### Fluorescence confocal microscopy.

HeLa cells were infected with strains containing pLW-*hns*p-*gfp* as described earlier. Supernatants were removed after infection, and the samples were washed three times and fixed with 4% (wt/vol) formaldehyde. DNA was visualized by Hoechst staining, and samples were mounted using ProLong Gold Antifade Mountant. DNA (Hoechst) and reporter activation (GFP) were imaged with a model TCS SP8 confocal fluorescence microscope (Leica, Wetzlar, Germany), and GFP fluorescence intensities of attached or free bacteria were then measured with ImageJ software (NIH, Bethesda, MD, USA). Three independent experiments were performed.

### TEM.

The collected unattached and attached bacteria were fixed as previously described (18). In brief, both unattached and attached bacteria were harvested and fixed with a solution of 2.5% (vol/vol) glutaraldehyde and 1% osmium tetroxide. The samples were then dehydrated through a series of ethanol solutions, embedded in LR white resin, cut into ultrathin sections 70 nm in thickness, and imaged by TEM using a model HT7700 microscope (Hitachi, Tokyo, Japan). Cross sections with similar diameters and clear cell walls were included in the analysis. Measurements were recorded at intervals of 10° around each bacterial cell. Positions with ambiguous cell membrane or ambiguous exterior electron-dense material boundary were neglected. Approximately 15 to 25 valid positions per cross section were measured. Ten cross sections from each group were analyzed. The data were analyzed and presented as notched box plots using an online tool (60).

### Statistical analyses.

Data are presented as the mean ± standard deviation (SD) of at least three independent replicates. Data from mouse survival passage experiments were analyzed for significant differences with the chi-squared test. Data from other animal experiments were analyzed with the Mann-Whitney *U* test on raw CFU values. Data of outer membrane material width measurements were visualized with notched box plots. In these box plots, the sample means with their 95% confidence intervals were also plotted, with the notch showing the 95% confidence interval for the median. In general, when notches do not overlap, the medians can be considered significantly different (61). For the rest of the experiments, differences between two groups were evaluated using a two-tailed Student’s *t* test. A *P* value of <0.05 was considered statistically significant. In the figures, asterisks indicate significant differences (*, *P* < 0.05; **, *P* < 0.01; ***, *P* < 0.001).
