# Supplementary figures and images for: Attachment of Enterohemorrhagic Escherichia coli to Host Cells Reduces O Antigen Chain Length at the Infection Site That Promotes Infection
Source: mBio. 2021 Dec 14;12(6):e02692-21. doi: 10.1128/mBio.02692-21 (PMC8669466; doi:10.1128/mBio.02692-21)

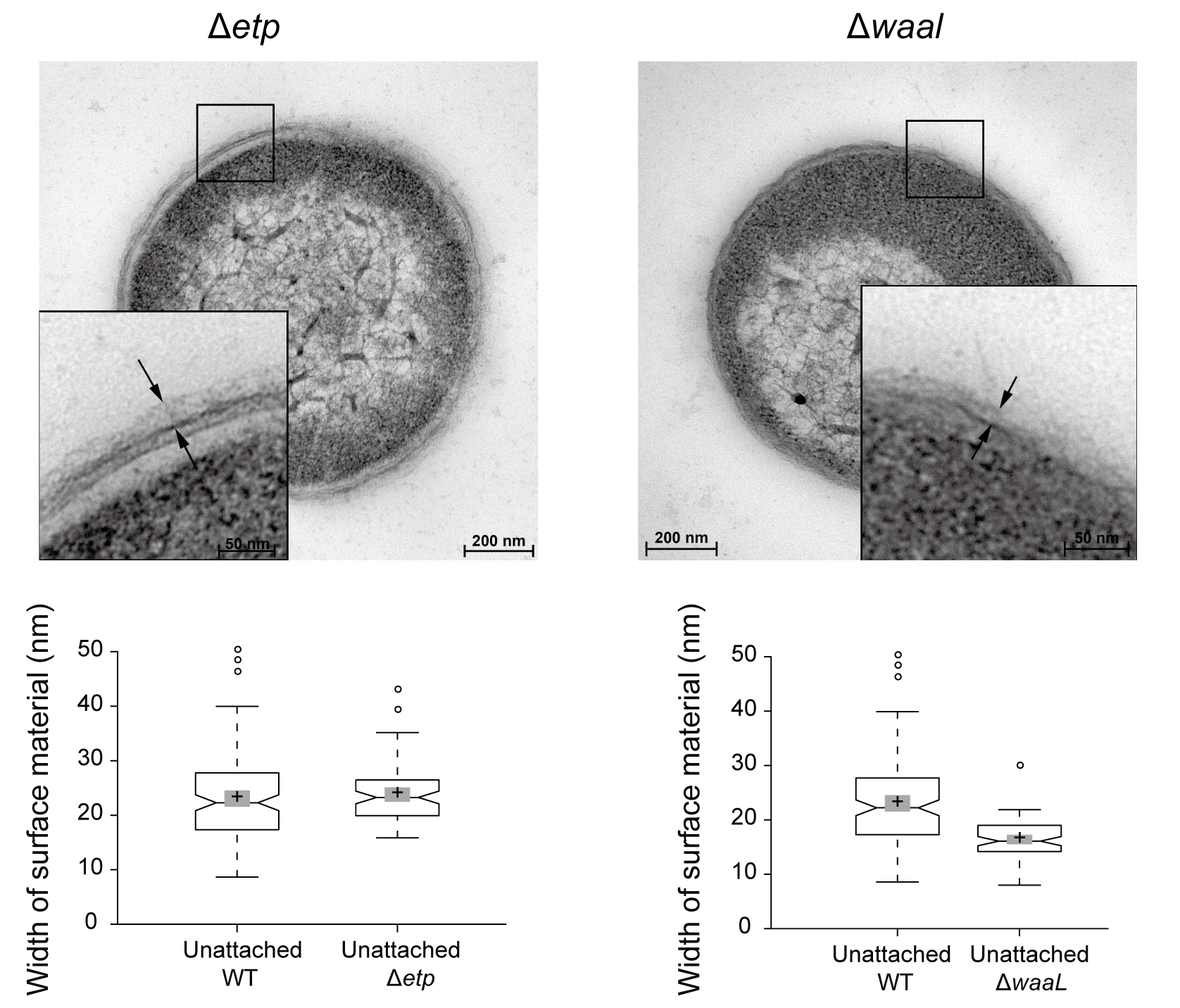

Supplement: FIG S1 [file mbio.02692-21-sf001.tif]

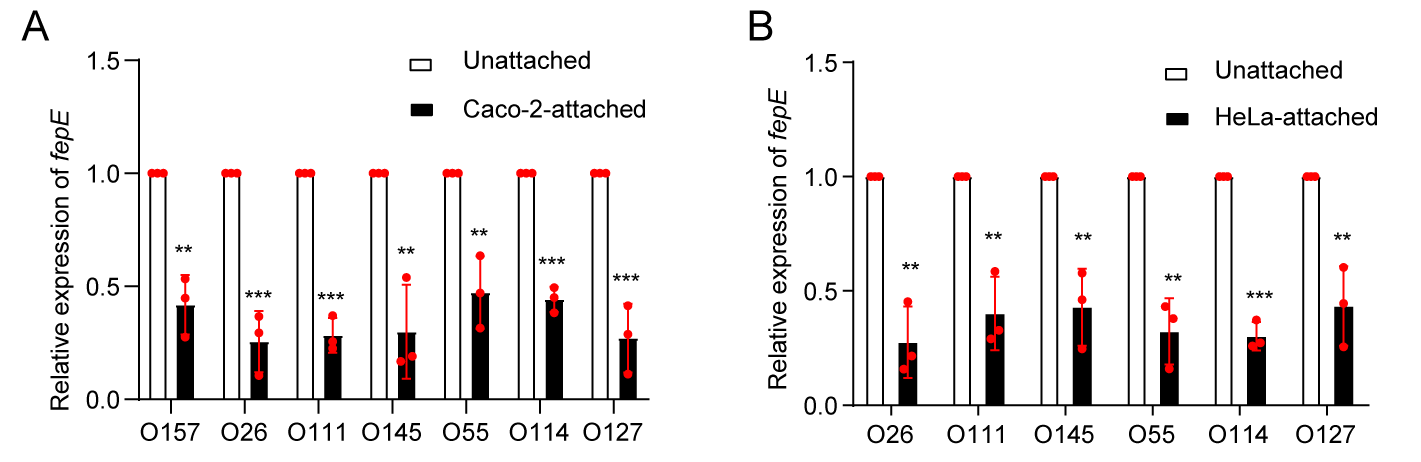

Supplement: FIG S2 [file mbio.02692-21-sf002.tif]

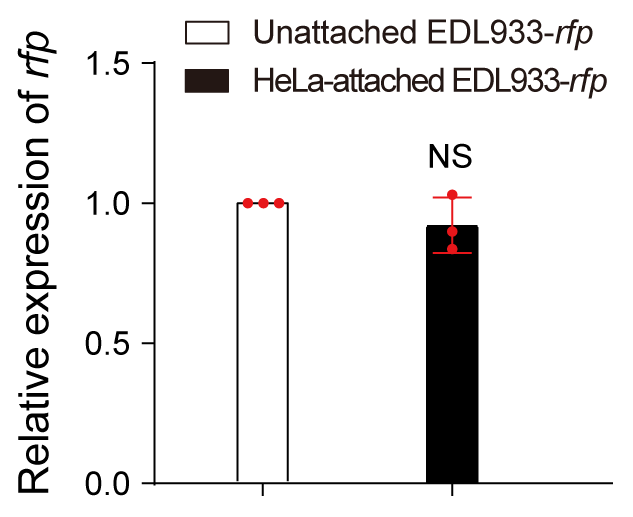

Supplement: FIG S3 [file mbio.02692-21-sf003.tif]

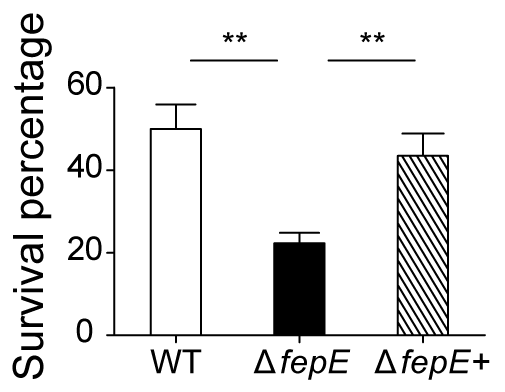

Supplement: FIG S4 [file mbio.02692-21-sf004.tif]

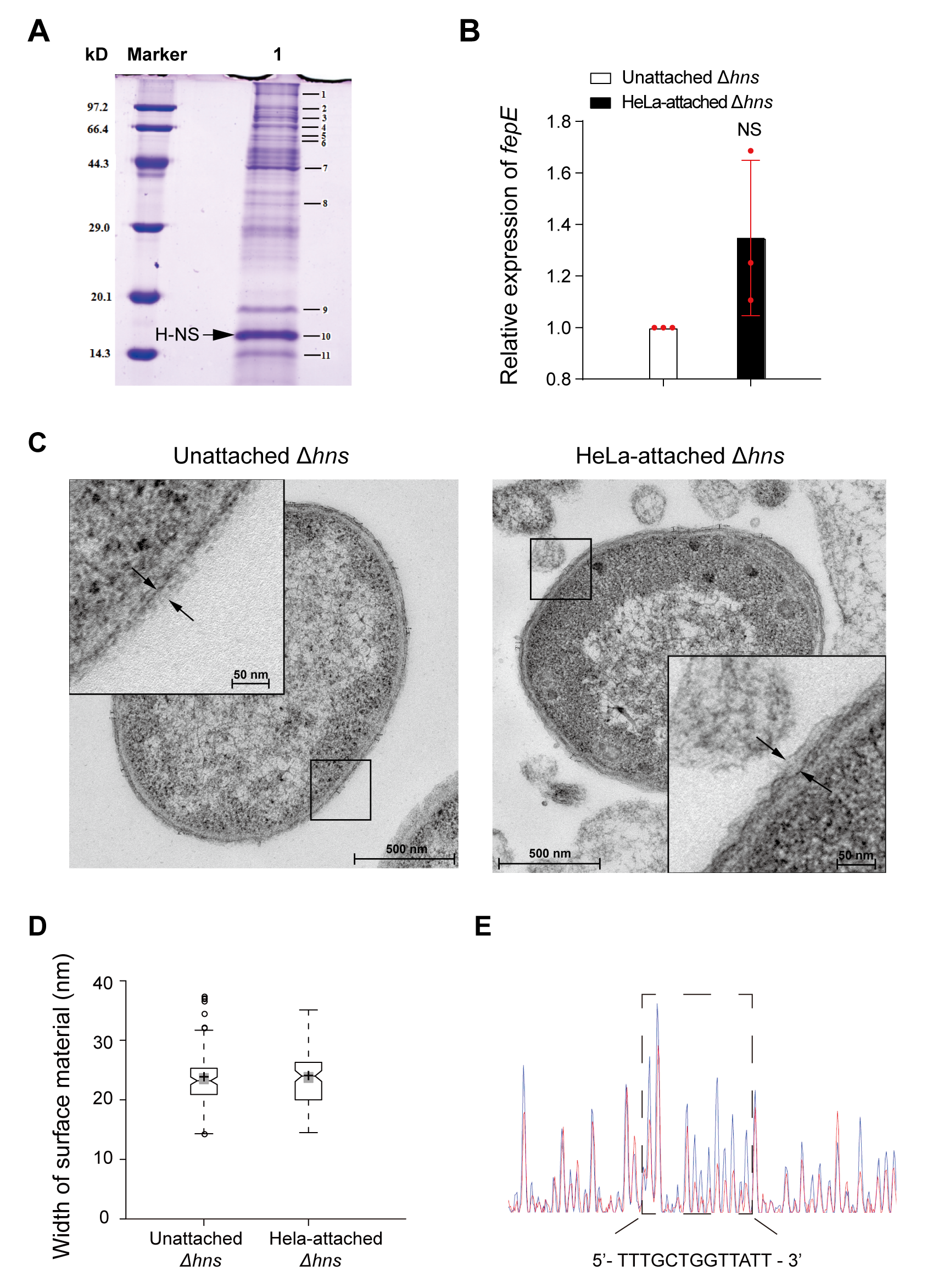

Supplement: FIG S5 [file mbio.02692-21-sf005.tif]

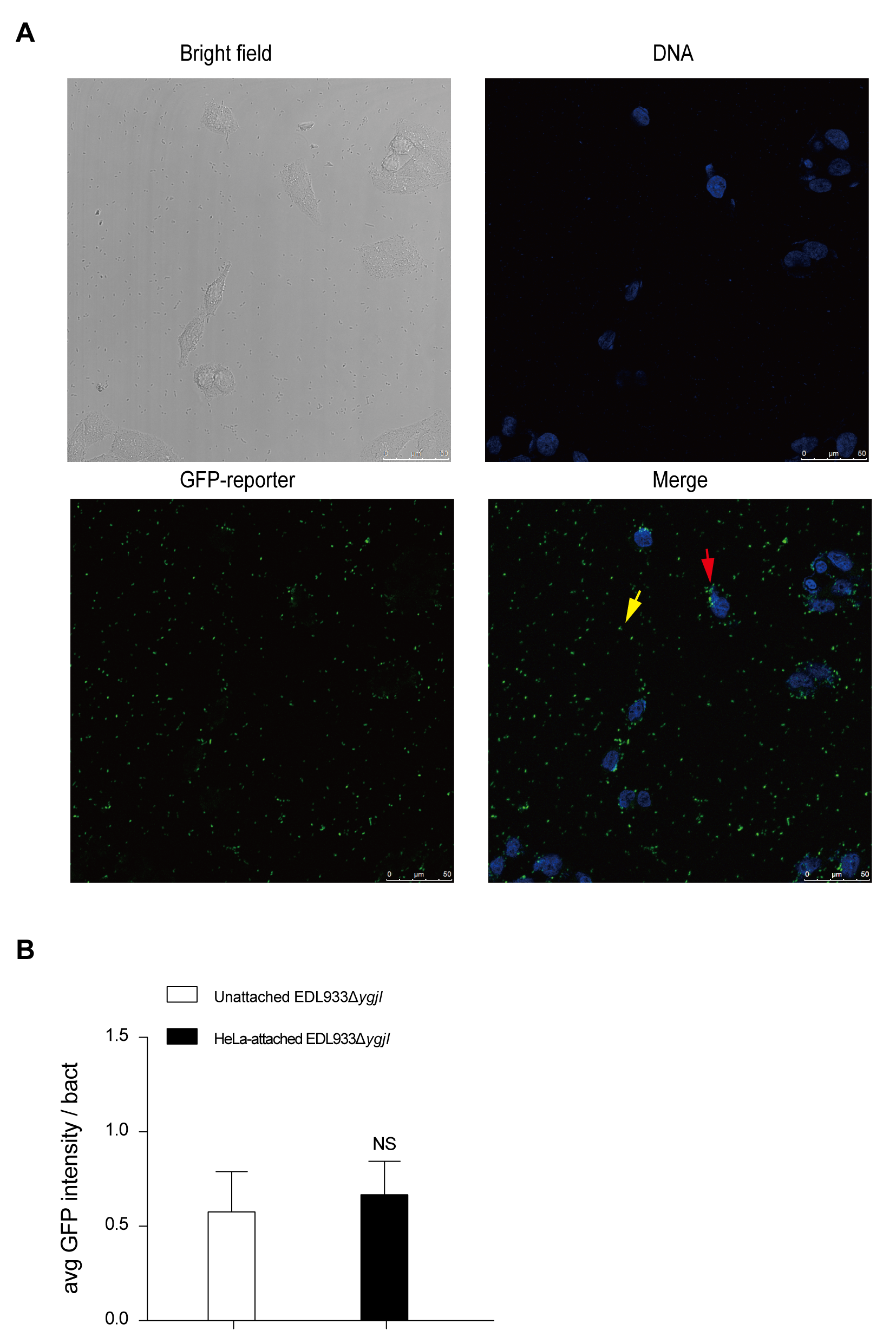

Supplement: FIG S6 [file mbio.02692-21-sf006.tif]
