# Supplementary material for: Attachment of Enterohemorrhagic Escherichia coli to Host Cells Reduces O Antigen Chain Length at the Infection Site That Promotes Infection
Source: mBio. 2021 Dec 14;12(6):e02692-21. doi: 10.1128/mBio.02692-21 (PMC8669466; doi:10.1128/mBio.02692-21)
Supplement: TABLE S1 [file mbio.02692-21-st001.docx]

**Table S1** Recovery of EDL933Δ*espAD*, EDL933Δ*fepE*Δ*espAD* and EDL933Δ*fepE+*Δ*espAD* from feces after oral administration of the EDL933 mutant strains.

| Strains | No. of mice with a positive sample; n = 4 mice/trial (total no. of positive mice/12 mice examined over three trials) | | |
| --- | --- | --- | --- |
|  | Trials | | |
|  | Ⅰ | Ⅱ | Ⅲ |
| EDL933Δ*espAD* | 4 | 3 | 3 (10) |
| EDL933Δ*fepE*Δ*espAD* | 2 | 1 | 1 (4)^*^ |
| EDL933Δ*fepE*+Δ*espAD* | 3 | 3 | 4 (10) |

^*^Significantly different (P < 0.05) from the EDL933Δ*espAD* and EDL933Δ*fepE*+Δ*espAD* mutant strain results.
