# Supplementary material for: Attachment of Enterohemorrhagic Escherichia coli to Host Cells Reduces O Antigen Chain Length at the Infection Site That Promotes Infection
Source: mBio. 2021 Dec 14;12(6):e02692-21. doi: 10.1128/mBio.02692-21 (PMC8669466; doi:10.1128/mBio.02692-21)
Supplement: TABLE S2 [file mbio.02692-21-st002.docx]

**Table S2** Proteins identified from DNA pulldown assay

| **Protein order** | **Protein description** |
| --- | --- |
| 1 | translation elongation factor Tu |
| 2 | pyruvate dehydrogenase |
| 3 | maltodextrin phosphorylase |
| 4 | *rpsA* gene product |
| 5 | GroEL |
| 6 | GroEL protein |
| 7 | *tuf* gene product |
| 8 | *cbpA* gene product |
| 9 | Dps |
| 10 | H-NS |
| 11 | C-lysozyme inhibitor |
