# Supplementary material for: Attachment of Enterohemorrhagic Escherichia coli to Host Cells Reduces O Antigen Chain Length at the Infection Site That Promotes Infection
Source: mBio. 2021 Dec 14;12(6):e02692-21. doi: 10.1128/mBio.02692-21 (PMC8669466; doi:10.1128/mBio.02692-21)
Supplement: TABLE S3 [file mbio.02692-21-st003.docx]

**Table S3** Gene mutants containing pLW-*hn*sp-*lux*, whose luminescence intensities were significantly altered compared with those of the EDL933 strain harboring pLW-*hns*p-*lux*

| **Gene order** | **Locus_tag** | **Product description** |
| --- | --- | --- |
| 1 | EDL933_3347 | ABC transporter ATP-binding protein YejF |
| 2 | EDL933_0318 | function unknown |
| 3 | EDL933_4797 | Serine transporter |
| 4 | EDL933_0364 | AidA-I adhesin-like protein |
| 5 | EDL933_3528 | LacY proton/sugar symporter |
| 6 | EDL933_0803 | Sodium:dicarboxylate symporter family |
| 7 | EDL933_2173 | MFS-1/Nitrate/nitrite transporter |
| 8 | EDL933_4300 | YgjI |
| 9 | EDL933_1450 | deacetylase PgaB |
