# Supplementary material for: Attachment of Enterohemorrhagic Escherichia coli to Host Cells Reduces O Antigen Chain Length at the Infection Site That Promotes Infection
Source: mBio. 2021 Dec 14;12(6):e02692-21. doi: 10.1128/mBio.02692-21 (PMC8669466; doi:10.1128/mBio.02692-21)
Supplement: TABLE S4 [file mbio.02692-21-st004.docx]

**Table S4** Strains, plasmids, and primers used in this study.

| **Strains** | | | | **Genotype or description** | **Source or reference** | | |
| --- | --- | --- | --- | --- | --- | --- | --- |
| EDL933 | | | | Wild-type *E*. *coli* O157: H7 strain EDL933 | Lab collection | | |
| EDL933Nal^R^ | | | | Wild-type *E*. *coli* O157: H7 strain EDL933 with resistance to nalidixic acid | This work | | |
| EDL933Δ*etp* | | | | *etp* mutant strain of EDL933 | This work | | |
| EDL933Δ*waaL* | | | | *waaL* mutant strain of EDL933 | This work | | |
| EDL933Δ*fepE* | | | | *fepE* mutant strain of EDL933 | This work | | |
| EDL933Δ*fepE*+ | | | | *fepE* complementary strain of EDL933Δ*fepE* carrying pLW2901 | This work | | |
| EDL933Δ*wzz* | | | | *wzz* mutant strain of EDL933 | This work | | |
| EDL933-*rfp* | | | | EDL933 strain with *rfp* constitutively expressed in the genome | This work | | |
| EDL933-*fepE*++ | | | | *fepE* overexpression strain of EDL933 carrying pLW2904 | This work | | |
| EDL933Δ*hns* | | | | *hns* mutant strain of EDL933 | This work | | |
| EDL933Δ*hns*+ | | | | *hns* complementary strain of EDL933Δ*hns* carrying pLW2902 | This work | | |
| EDL933Δ*espAD* | | | | *espAD* mutant strain of EDL933 | This work | | |
| EDL933Δ*fepE*Δ*espAD* | | | | *espAD* mutant strain of EDL933Δ*fepE* | This work | | |
| EDL933Δ*fepE*+Δ*espAD* | | | | EDL933Δ*fepE*Δ*espAD* mutant carrying pLW2901 | This work | | |
| EDL933Δ*ygjI* | | | | *ygjI* mutant strain of EDL933 | This work | | |
| EDL933Δ*ygjI*+ | | | | *ygjI* complementary strain of EDL933Δ*ygjI* carrying pLW2903 | This work | | |
| EDL933Δ*fepE*-*bs* | | | | mutant strain of EDL933 with major binding site of H-NS in promoter region of *fepE* deleted | This work | | |
| EDL933Δ*fepE*-*bs*Δ*ygjI* | | | | *ygjI* mutant strain of EDL933Δ*fepE*-*bs* | This work | | |
| G4958 | | | | Wild-type EHEC O26: H11 | Lab collection | | |
| G4947 | | | | Wild-type EHEC O111: H8 | Lab collection | | |
| G1345 | | | | Wild-type EHEC O145: H28 | Lab collection | | |
| G4944 | | | | Wild-type EPEC O55: H7 | Lab collection | | |
| G1179 | | | | Wild-type EPEC O114: H2 | Lab collection | | |
| G1094 | | | | Wild-type EPEC O127: H7 | Lab collection | | |
| **Plasmids** | | | | | | | |
| pTrc99a | | | | Expression vector, AmpR | Lab collection | | |
| pACYC184 | | | | Expression vector, CmR, TcR | Lab collection | | |
| pETDuet-1 | | | | Expression vector, AmpR | Lab collection | | |
| pLW2901 | | | | pACYC184 carrying *fepE* with its native promoter | Lab collection | | |
| pLW2902 | | | | pACYC184 carrying *hns* with its native promoter | Lab collection | | |
| pLW2903 | | | | pACYC184 carrying *ygjI* with its native promoter | Lab collection | | |
| pLW2904 | | | | pTrc99a carrying *fepE* fused with a strong promoter | Lab collection | | |
| pLW-*fepE*p-*gfp* | | | | pETDuet-1 carrying *fepE* promoter fused GFP, AmpR, KanR | This work | | |
| pLW-*hns*p-*gfp* | | | | pETDuet-1 carrying *hns* promoter fused GFP, AmpR, KanR | This work | | |
| pCX340 | | | | cloning vector used to fuse effectors to the mature form of Bla-1 β-lactamase | ATCC | | |
| pLW-*map*-Bla | | | | pCX340 carrying *map* from O157 | This work | | |
| pLW-*hns*p-*lux* | | | | *hns* promoter fused to *lux* reporter gene | This work | | |
| **Primers** | | | | | | | |
| Primers for gene mutation | | | | | | | |
| *fepE* | F | GTGGAGATTTCCCCCTATTGGACTCATTTTCAGGTTATGTGTAGGCTGGAGCTGCTTCG | | | | | |
| *fepE* | R | ATCAGCGCGGAAAGGATCACAATAATCGCCTTACCCGGACATATGAATATCCTCCTTAG | | | | | |
| *etp* | F | GTGGATTCAAACATGGCCCAACTAAAATTTAACTCAATCGTGTAGGCTGGAGCTGCTTCG | | | | | |
| *etp* | R | TTACCGGCTGAGGCGCTTCGCCCATTCCTGACTGGCGCCATATGAATATCCTCCTTAG | | | | | |
| *waaL* | F | ATGACCTCAACATTATTTTTCTCTCTCGAGAAAAAAAACGTGTAGGCTGGAGCTGCTTCG | | | | | |
| *waaL* | R | TTACTTGTTTTTCATCGCTAATAATAAGCCGGCGTAAACGCATATGAATATCCTCCTTAG | | | | | |
| *hns* | F | TCAACAAACCACCCCAATATAAGTTTGAGATTACTACAGTGTAGGCTGGAGCTGCTTCG | | | | |  |
| *hns* | R | CCGATGGCGGGATTTTAAGCAAGTGCAATCTACAAAAGACATATGAATATCCTCCTTAG | | | | |  |
| *ygjI* | F | | GAGATGTGTTATGTCTGATACCAAACGTAATACAATCGGGTGTAGGCTGGAGCTGCTTC | | |  |  |
| *ygjI* | R | | GAAGAGGGAAAATGCCTGCTCATTTTTTATTAGTCTGTTATGGGAATTAGCCATGGTCC | | |  |  |
| *espAD* | F | | TTTTGTTTTCCTGAGAAAAATTATCAAGAGGTATATAGGTGTAGGCTGGAGCTGCTTCG | | |  |  |
| *espAD* | R | | TATTATCAATAGTATTCATAATAAAATTCTCTTTAATAACATATGAATATCCTCCTTAG | | |  |  |
| *fepE-bs* | F1 | | AGTTGTTTACCACCATTGAAGG | | |  |  |
| *fepE-bs* | R1 | | GAATGCAACATTCACTCTATTTAAGAACATAATTATTTCCTGCAATTATGGGGTTATAGATAATGTTAATTTATCTGTTTAGCGTT | | |  |  |
| *fepE-bs* | F2 | | AACGCTAAACAGATAAATTAACATTATCTATAACCCCATAATTGCAGGAAATAATTATGTTCTTAAATAGAGTGAATGTTGCATTC | | |  |  |
| *fepE-bs* | R2 | | CGAAGCAGCTCCAGCCTACACTTAAACTAAGTGGTCTGCCATC | | |  |  |
| *fepE-bs* | F3 | | GATGGCAGACCACTTAGTTTAAGTGTAGGCTGGAGCTGCTTCG | | |  |  |
| *fepE-bs* | R3 | | ATCATGGCATCCTGTTTTCTCATATGAATATCCTTAG | | |  |  |
| *fepE-bs* | F4 | | CTAAGGAGGATATTCATATGAGAAAACAGGATGCCATGAT | | |  |  |
| *fepE-bs* | R4 | | GAAGAGGCGGTAACGAAAG | | |  |  |
| Primers for gene cloning | | | | | |  |  |
| Δ*fepE*+ | F | | CGGAATTCGTCGCCCAGTAATAATGAAAT | | |  |  |
| Δ*fepE*+ | R | | CATGCCATGGGATTAAACTAAGTGGTCTGCCATCA | | |  |  |
| *fepE* ++ | F | | CGGAATTCATGTCATCACTGAATATTAAACA | | |  |  |
| *fepE* ++ | R | | CGGGATCCTTAAACTAAGTGGTCTGCCATCA | | |  |  |
| Δ*hns*+ | F | | CGGAATTCATGAGCGAAGCACTTAAAATTCTG | | |  |  |
| Δ*hns*+ | R | | CATGCCATGGGATTATTGCTTGATCAGGAAATCGTCG | | |  |  |
| Δ*ygjI*+ | F | | CGGAATTCATGTCTGATACCAAACGTAATACAAT | | |  |  |
| Δ*ygjI*+ | R | | CATGCCATGGGAACGCCAGCCAGCAATGTTGATTAA | | |  |  |
| *hns* p | F | | CCCAAGCTTGGCTGGAGTTTATCATAATTCG | | |  |  |
| *hns* p | R | | TGAAAAGTTCTTCTCCTTTACTCATTGTAGTAATCTCAAACTTATATTGGG | | |  |  |
| *gfp* | F | | CCCAATATAAGTTTGAGATTACTACAATGAGTAAAGGAGAAGAACTTTTCA | | |  |  |
| *gfp* | R | | AAAACTGCAGTTATTTGTATAGTTCATCCATGCC | | |  |  |
| *fepE* p | F | | AAAACTGCAGTCTCTCCAGGGGCATTTG | | |  |  |
| *fepE* p | R | | TGAAAAGTTCTTCTCCTTTACTCATGGCAAACGCAAAAACGAC | | |  |  |
| *gfp*’ | F | | GTCCTTTTTGCGTTTGCCATGAGTAAAGGAGAAGAACTTTTCA | | |  |  |
| *gfp*’ | R | | CCCAAGCTT TTATTTGTATAGTTCATCCATGCC | | |  |  |
| *rfp* | F1 | | TATTATTCATTTGCTGAACGAAGGGCTGCCTTTTGGCGGCCCTTTTACTTTTGACGGCTAGCTCAGTCCTAGGTACAGTGCTAGCAAGGAGATATACTATGGCCTCCTCCGAGAAC | | |  |  |
| *rfp* | R1 | | CGAAGCAGCTCCAGCCTACACCTACAGGAACAGGTGGTGGC | | |  |  |
| *rfp* | F2 | | GCCACCACCTGTTCCTGTAGGTGTAGGCTGGAGCTGCTTCG | | |  |  |
| *rfp* | R2 | | TTACAGTGATAATTTATTATCACACATTGTTACATAGTTAAATAAAATCATATGAATATCCTCCTTAG | | |  |  |
| Primers for qRT-PCR | | | | | |  |  |
| *gyrB* | F | | ATGAAGGCGGCATCAAAG | | |  |  |
| *gyrB* | R | | CGCTGCGGAATGTTGTT | | |  |  |
| *fepE* | F | | GGTTGATGACAATGCCAGTAAGA | | |  |  |
| *fepE* | R | | GCGGTCCTGAGCCAGTTTT | | |  |  |
| *wzz* | F | | GCAGTATGCGAATCAGGCG | | |  |  |
| *wzz* | R | | GAGAACACCAACGGACGGG | | |  |  |
| *hns* | F | | GACGGTATTGACCCGAACGA | | |  |  |
| *hns* | R | | CCTTGCTCATCCATTGCTTTT | | |  |  |
| *rfp* | F | | CCACAACGAGGACTACACCA | | |  |  |
| *rfp* | R | | ATGCCGCTGGCGATTC | | |  |  |
| Primers for DNA pull down | | | | | |  |  |
| *fepE* p | F | | TCTCTCCAGGGGCATTTG | | |  |  |
| *fepE* p | R | | GGCAAACGCAAAAACGAC | | |  |  |
| Primers for EMSA | | | | | |  |  |
| *lacZ* | F | | CCGTTTCGCTGGTGGTC | | |  |  |
| *lacZ* | R | | TGAACTACCGCGACTGGAGA | | |  |  |
| *fepE* p | F | | TCTCTCCAGGGGCATTTG | | |  |  |
| *fepE* p | R | | GGCAAACGCAAAAACGAC | | |  |  |
